# **Bn_2_DT3A**, a Chelator for ^68^Ga Positron Emission Tomography: Hydroxide Coordination Increases Biological Stability of [^68^Ga][Ga(Bn_2_DT3A)(OH)]^−^

**DOI:** 10.1021/acs.inorgchem.2c01992

**Published:** 2022-10-17

**Authors:** Thomas W. Price, Isaline Renard, Timothy J. Prior, Vojtěch Kubíček, David M. Benoit, Stephen J. Archibald, Anne-Marie Seymour, Petr Hermann, Graeme J. Stasiuk

**Affiliations:** † Department of Imaging Chemistry and Biology, School of Biomedical Engineering and Imaging Sciences, King’s College London, London SE1 7EH, U.K.; ‡ Department of Biomedical Sciences, University of Hull, Cottingham Road, Hull HU6 7RX, U.K.; § Positron Emission Tomography Research Center, University of Hull, Cottingham Road, Hull HU6 7RX, U.K.; ∥ Chemistry, University of Hull, Cottingham Road, Hull HU6 7RX, U.K.; ⊥ Department of Inorganic Chemistry, Faculty of Science, Charles University, Hlavova 2030, Prague 2, Czech Republic; # E.A. Milne Centre for Astrophysics, Department of Physics and Mathematics, University of Hull, Cottingham Road, Hull HU6 7RX, U.K.

## Abstract

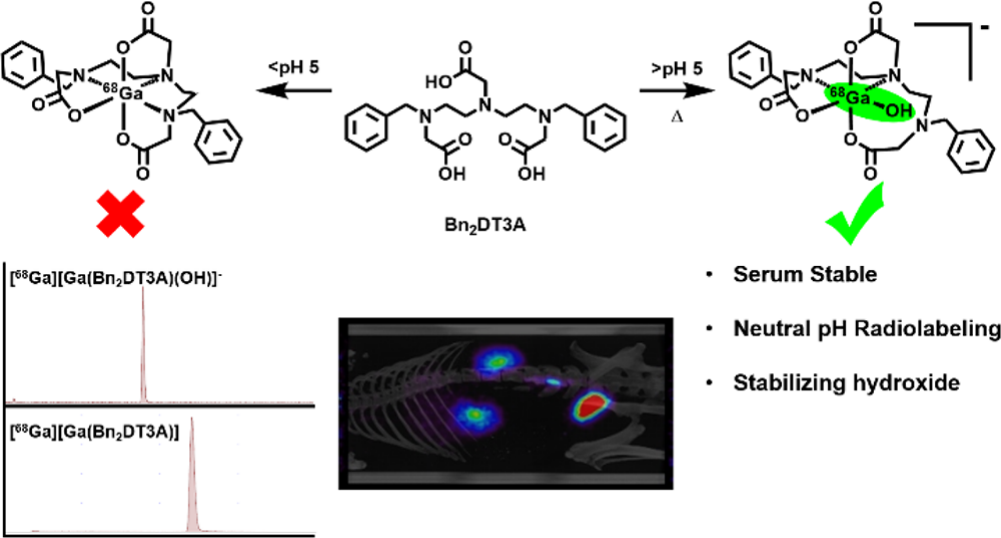

The chelator **Bn_2_DT3A** was used to produce a novel ^68^Ga complex for positron emission tomography (PET). Unusually, this system is stabilized by a coordinated hydroxide in aqueous solutions above pH 5, which confers sufficient stability for it to be used for PET. **Bn_2_DT3A** complexes Ga^3+^ in a hexadentate manner, forming a *mer-mer* complex with log *K*([Ga(**Bn_2_DT3A**)]) = 18.25. Above pH 5, the hydroxide ion coordinates the Ga^3+^ ion following dissociation of a coordinated amine. **Bn_2_DT3A** radiolabeling displayed a pH-dependent speciation, with [^68^Ga][Ga(**Bn_2_DT3A**)(OH)]^−^ being formed above pH 5 and efficiently radiolabeled at pH 7.4. Surprisingly, [^68^Ga][Ga(**Bn_2_DT3A**)(OH)]^−^ was found to show an increased stability *in vitro* (for over 2 h in fetal bovine serum) compared to [^68^Ga][Ga(**Bn_2_DT3A**)]. The biodistribution of [^68^Ga][Ga(**Bn_2_DT3A**)(OH)]^−^ in healthy rats showed rapid clearance and excretion *via* the kidneys, with no uptake seen in the lungs or bones.

## Abstract 2

The chelator **Bn_2_DT3A** produces a novel ^68^Ga complex for positron emission tomography (PET) imaging. This complex is stabilized by a coordinated hydroxide in aqueous solutions above pH 5. Efficient radiolabeling at pH 7.4 gives [^68^Ga][Ga(**Bn_2_DT3A**)(OH)]^−^, which was found to be stable in FBS for over 2 h. The biodistribution of [^68^Ga][Ga(**Bn_2_DT3A**)(OH)]^−^ shows rapid clearance and excretion *via* the kidneys, showing that **Bn_2_DT3A** has excellent properties as a chelator for ^68^Ga PET tracers.

## Introduction

Positron emission tomography (PET) is a highly sensitive technique that can be used to image molecular processes.^1^ While the resolution is not as high as other imaging modalities (typically in the mm range),^1^ the high sensitivity allows for target-specific imaging of cellular receptors using peptides and antibodies.^2^

A range of radioactive nuclei can be used for PET;^2^ gallium-68 (^68^Ga) is a PET isotope that has favorable physical decay properties for diagnostic imaging,^3,4^ with a high positron branching ratio (β^*+*^ = 89%) and a half-life (τ_1/2_ = 67.71 min) suitable for use with small peptide targeting units.^2−4^^68^Ga is also available from a radionuclide generator.^4^ This is a more accessible route to on-site isotope production than the more conventional cyclotron production, although the activities produced are lower than those achievable by cyclotron production of ^68^Ga.^5−7^

While weakly coordinated Ga^3+^ salts such as gallium citrate or nitrate have been used in clinical nuclear imaging,^5^ to achieve more specific images of disease, ^68^Ga is typically incorporated into a radiotracer through the use of a chelator.^5,8^ These radiotracers have found significant success in recent years, in particular the somatostatin targeting [^68^Ga][Ga(**DOTATATE**)], which has been approved for diagnostic imaging of neuroendocrine tumors^9,10^ and prostate specific membrane antigen targeting ^68^Ga probes, which are being utilized clinically for identification of prostate cancer metastases.^11−15^

A range of chelators have been applied to ^68^Ga complexation;^8,16^ the most widely used is the macrocycle 1,4,7,10-tetraazacyclododecane-1,4,7,10-tetraacetic acid (**DOTA**, Figure S1).^5,16^**DOTA** is a versatile chelator, capable of complexing a variety of metals.^5^ However, this versatility also means that it is not the ideal chelator for ^68^Ga.^16^ This is reflected in the forcing radiolabeling conditions required for radiochemical yields (RCYs) >95% (elevated temperatures of 80 °C and acidic conditions of pH 4)^17,18^ and reduced stability of the resulting complex (80% intact after 2 h incubation in serum).^19^ A more suitable macrocyclic chelator, 1,4,7-triazacyclononane-1,4,7-triacetic acid (**NOTA**, Figure S1), demonstrates the improved radiolabeling efficiency (no heating required)^16−18,20^ and stability (>98% stable to serum over 2 h)^19,20^ that can be obtained by using specifically designed chelators for ^68^Ga.

An area of growing interest in the development of chelators for ^68^Ga is the ability to radiolabel the chelator at higher pH values.^8,21,22^ This would allow for a simpler radiolabeling process and for the use of targeting moieties that may degrade under acidic conditions, expanding the breadth of diagnostic agents possible using ^68^Ga.^22^ Some key examples of chelators that have achieved this goal are 4-acetamido-*N*^1^,*N*^7^-bis[(3-hydroxy-1,6-dimethyl-4-oxo-1,4-dihydropyridin-2-yl)methyl]-4-(3-{[(3-hydroxy-1,6-dimethyl-4-oxo-1,4-dihydropyridin-2-yl)methyl]amino}-3-oxopropyl)heptanediamide (**THP**, Figure S1),^23,24^ 2,2′-{6-[(1-carboxyethyl)amino]-6-phenyl-1,4-diazepane-1,4-diyl}dipropionic acid (**DATA^PPh^**, Figure S1),^25^ and 2,2′-{ethane-1,2-diylbis[(2-hydroxybenzyl)azanediyl]}diacetic acid (**HBED**, Figure S1).^12,22,26^ These chelators have an acyclic or semicyclic design that improves the coordination kinetics.

Substitution of chelators can impact upon the biodistribution of PET radiotracers;^27,28^ as such, having a range of suitable chelators will aid in the rapid development of a radiotracer with optimized biodistribution and target uptake. Further development of chelators for ^68^Ga will also aid in the understanding of the design of systems capable of producing highly stable chelates under mild conditions. This would allow for the radiolabeling of sensitive biomolecules possessing an appropriate biological half-life.

**Scheme 1:**
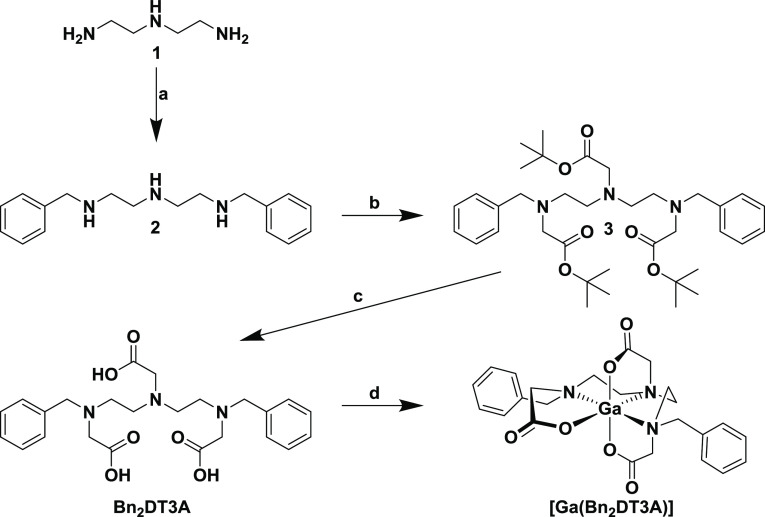
Synthesis of Bn_2_DT3A and Subsequent Complexation of Ga^3+^. (a) (i) EtOH, Benzaldehyde, Reflux. (ii) NaBH_4_, 0 °C. (b) MeCN, Na_2_CO_3_, *tert*-Butyl Bromoacetate, 60 °C. (c) DCM, TFA. 0 °C to RT. (d) H_2_O, GaCl_3_, pH 4, Reflux

In this manuscript, we report the synthesis of a novel hexadentate acyclic chelator, 2,2′-({[(carboxymethyl)azanediyl]bis(ethane-2,1-diyl)}bis[benzylazanediyl])-diacetic acid (**Bn_2_DT3A**, Scheme 1), characterize its Ga^3+^ complex, and explore the radiolabeling efficiency of this system with ^68^Ga. **Bn_2_DT3A** resembles the well-studied diethylenetriamine-*N*,*N*,*N*′,*N*″,*N*″-pentaacetic acid (**DTPA**, Figure S1) chelator; however, benzyl units have been substituted in place of two of the acetic acid arms. While **DTPA** has been applied to ^68^Ga complexation, the radiolabeling efficiency was not sufficiently high^29,30^ and the resulting complex was unstable under relevant biological conditions.^29,30^ A more rigid derivative, 2,2′-{[2-({2-[bis(carboxymethyl)amino]cyclohexyl}[carboxymethyl]-amino)ethyl]azanediyl}diacetic acid (**CHX-A″-DTPA**, Figure S1), has been demonstrated to produce a stable complex with ^68^Ga^31,32^ and applied to imaging in humans.^32^ The substitution of the acetate arms of **DTPA** for benzyl units to give **Bn_2_DT3A** results in a chelator with a coordination number that matches the ideal octahedral Ga^3+^ coordination sphere (coordination number = 6),^33^ increases the ligand rigidity, and offers sites distant from the coordination sites for future functionalization. Benzyl units were chosen to increase steric bulk and lipophilicity and therefore reduce access of competitors to the Ga^3+^ ion. The benzyl units also afforded a UV-active tag to aid in monitoring synthesis and purification.

Upon investigation of this system, we demonstrated that a species with a hydroxide anion coordinated to the Ga^3+^ center was present when the complex was formed under neutral conditions. We have shown *via* computational studies that the coordination of a hydroxide anion to the Ga^3+^ center of Ga-**Bn_2_DT3A** results in a system with a larger energy barrier to dissociation than the equivalent water complex. This is reflected in the *in vitro* stability to FBS where the hydroxide complex is stable for over 2 h.

## Results and Discussion

### Ligand Synthesis and Ga^3+^ Complexation

**Bn_2_DT3A** was prepared in a three-step synthesis (Scheme 1), with an overall yield of 23%. Diethylenetriamine was selectively protected at the terminal amine sites through a reductive amination with benzaldehyde.^34^ This selective protection is confirmed by the symmetry of the benzyl arms and alkyl backbone in the ^1^H NMR (Figure S2). Alkylation with *tert*-butyl bromoacetate introduced protected carboxylic acid moieties to yield the proligand, **3**.^35^ The incorporation of the acetate arms in two different environments can be seen in the ^1^H NMR, reflecting the central and terminal amine functionalities (Figure S6). The proligand was then deprotected using trifluoroacetic acid to yield the ligand **Bn_2_DT3A** as a white powder. The benzyl units are retained, and the two acetate arm environments are distinguishable in the ^1^H NMR with the central arm being more shielded than the terminal arms (Figure S10).

Complexation of Ga^3+^ by **Bn_2_DT3A** was achieved at room temperature and confirmed by HRMS (*m*/*z* = 524.1734, calculated *m*/*z* = 524.1307). The ^1^H NMR indicates a high level of asymmetry with multiple overlapping peaks with a high degree of ^1^H–^1^H coupling, limiting the analysis (Figure S14). Regardless, the spectra confirm the suggested model as well-resolved spectra were obtained in the pH region in which [M(L)] is the dominant species present in solution. At pD 3.3, the presence of two sharp peaks, at 3.56 and 3.85 ppm, seem to correspond to the formation of the protonated species, [Ga(HL)]^+^, although this could not be confirmed due to overlap with the surrounding peaks. While there are clearly changes in the spectra between pD 4.0 and pD 7.3, such as the broadening of the signal between 3.45 and 3.33 ppm and the change in spectral form at 3.06 ppm, these are difficult to quantify due to the large number of overlapping signals, making precise analysis unsuitable. Hydroxide coordination leads to significant signal broadening in spectra collected above pD 6.8, which could be ascribed to intermediate ligand flexibility of the partly coordinated ligand molecule. In addition, decomplexation can be seen at high pH by the improved resolution of the ^1^H NMR spectrum reflecting the free ligand being produced, increasing symmetry and flexibility resulting in sharp, well-defined peaks being observed at pD 8.8 and 10.0 (Figure S18).

### Crystal Structure

**Figure 1:**
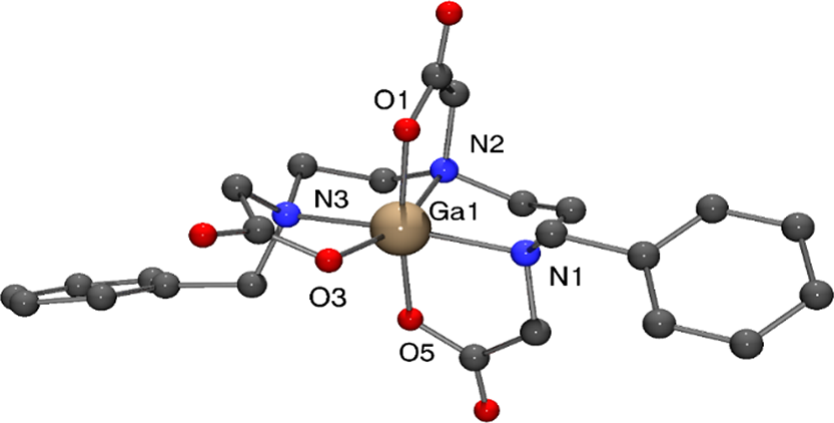
Molecular structure of [Ga(**Bn_2_DT3A**)] determined by X-ray crystallography. Hydrogen atoms have been omitted for clarity. Colors: gallium (pale brown); carbon (gray); nitrogen (blue); oxygen (red).

A crystal of suitable quality for single-crystal X-ray diffraction was obtained from an acidic aqueous solution of [Ga(**Bn_2_DT3A**)]. The obtained structure (Figure 1) shows a hexadentate ligand, fully satisfying the coordination sphere of Ga^3+^. The nitrogen atoms coordinate Ga^3+^ in a *mer* fashion, as do the oxygen atoms of the carboxylate arms. The Ga^3+^ ion lies 0.220(5) Å above the plane of the three nitrogen atoms. The bite angle of each chelating unit is between 80.9(3)° and 87.0(3)°. These angles are comparable to those reported for [Ga(**DOTA**)]^−^,^36^ [Ga(**NOTA**)],^37^ and [Ga(**EDTA**)]^−^ (**EDTA** = ethylenediamine-*N*,*N*,*N*′,*N*′-tetraacetic acid, Figure S1).^38^ The face where the two terminal ends of the ligand meet is slightly open in comparison to the other faces of the distorted octahedral geometry; the angle between N1 and O3 is 108.8(1)°. There is also a degree of asymmetry in the O-Ga-O angles (O1-Ga-O3 = 87.1(3)°, O3-Ga-O5 = 99.4(3)°) that is not seen in the crystal structures of the macrocyclic Ga^3+^ complexes but was also reported for [Ga(**EDTA**)]^−^.^38^ The Ga1–N2 bond length (2.077(6) Å) is a little shorter than those to N1 and N3 (2.120(7) and 2.129(8) Å, respectively). This may reflect the strain induced by coordination of the central amine to Ga^3+^; this strain has previously been reported to prevent the coordination of the central amine in tripodal chelates with Ga^3+^.^19,39^ The gallium-to-oxygen bond lengths are shorter and lie in the range 1.937(5) to 1.987(5) Å, comparable to those reported for [Ga(**DOTA**)]^−^,^36^ [Ga(**NOTA**)],^37^ and [Ga(**EDTA**)]^−^.^38^ The Ga^3+^ complex does not form any classical hydrogen bonds, but within the solid-state structure, there are many C–H···O interactions. Further details of the crystal structure determination are given in the SI (Figure S24 and Table S2). Crystals were grown at two further pH levels (5.3 and 6.8): the crystal structure obtained from these two preparations was the same; the same molecule, [Ga(**Bn_2_DT3A**)], was present as a more complicated hydrate. This is likely due to the low solubility of the neutral complex in comparison to other species in solution. Further details are given in the SI (Figures S25 and S26, Table S3).

### Thermodynamic Stability

Potentiometry was performed to obtain protonation constants of **Bn_2_DT3A** and on the system with Ga^3+^, Cu^2+^, and Zn^2+^ to obtain thermodynamic stability constants and an understanding of the effect of pH on speciation in solution. Five protonation constants were determined for **Bn_2_DT3A** (Table 1). By comparison to similar ligands (Table 1),^40−45^ the first three protonation constants were assigned to the amines of **Bn_2_DT3A**. The remaining two protonation constants correspond to the carboxylic acid arms, with the final arm being too acidic to detect the corresponding constant. The amine sites are more acidic than those reported for **DTPA **— this is due to the stabilizing effect of the additional negatively charged carboxylate arms in **DTPA**, making amine deprotonation more difficult.^40^ This can be seen by comparing the reported values for the protonation constants of glycine (log *K_a_* = 9.8)^46^ and benzylamine (log *K_a_* = 9.36).^47^ Benzylamine has a more acidic amine than glycine as it lacks the internal hydrogen bonding provided by the carboxylate arm. The two carboxylate protonation constants obtained for **Bn_2_DT3A** have similar values—this contrasts with those reported for **NOTA**, which have significantly differing values. This is likely due to the flexibility of the linear ligand **Bn_2_DT3A** allowing for independent protonation of the arms, whereas in the rigid macrocyclic system of **NOTA**, the arms will likely interact, forming internal hydrogen bonds where a deprotonated arm stabilizes a protonated arm at low pH. It is surprising that the carboxylate arms of **Bn_2_DT3A** are approximately one log *K_a_* unit more acidic than those of **DTPA**, although this may again be due to hydrogen bonding between the additional carboxylate arms of **DTPA**, stabilizing the partially deprotonated ligand at low pH.^40^

**Table 1:** Protonation Constants of the Discussed Ligands

constant	**Bn_2_DT3A**^a^	**1**(41)	**DTPA**(40,41)	**NOTA**(44,45,48)
log *K*_1_	9.70	9.84	10.52	13.17
log *K*_2_	7.48	9.02	8.56	5.74
log *K*_3_	3.34	4.25	4.31	3.22
log *K*_4_	1.50		2.8	1.96
log *K*_5_	1.40		2.22	0.7

a *T* = 25 °C, *I* = 0.1 M NMe_4_Cl.

A 1:1 metal:ligand complex is formed between Ga^3+^ and **Bn_2_DT3A** between pH 2 and 8. Above this pH, the formation of [Ga(OH)_4_]^−^ dominates the speciation of Ga^3+^ in solution.

**Figure 2:**
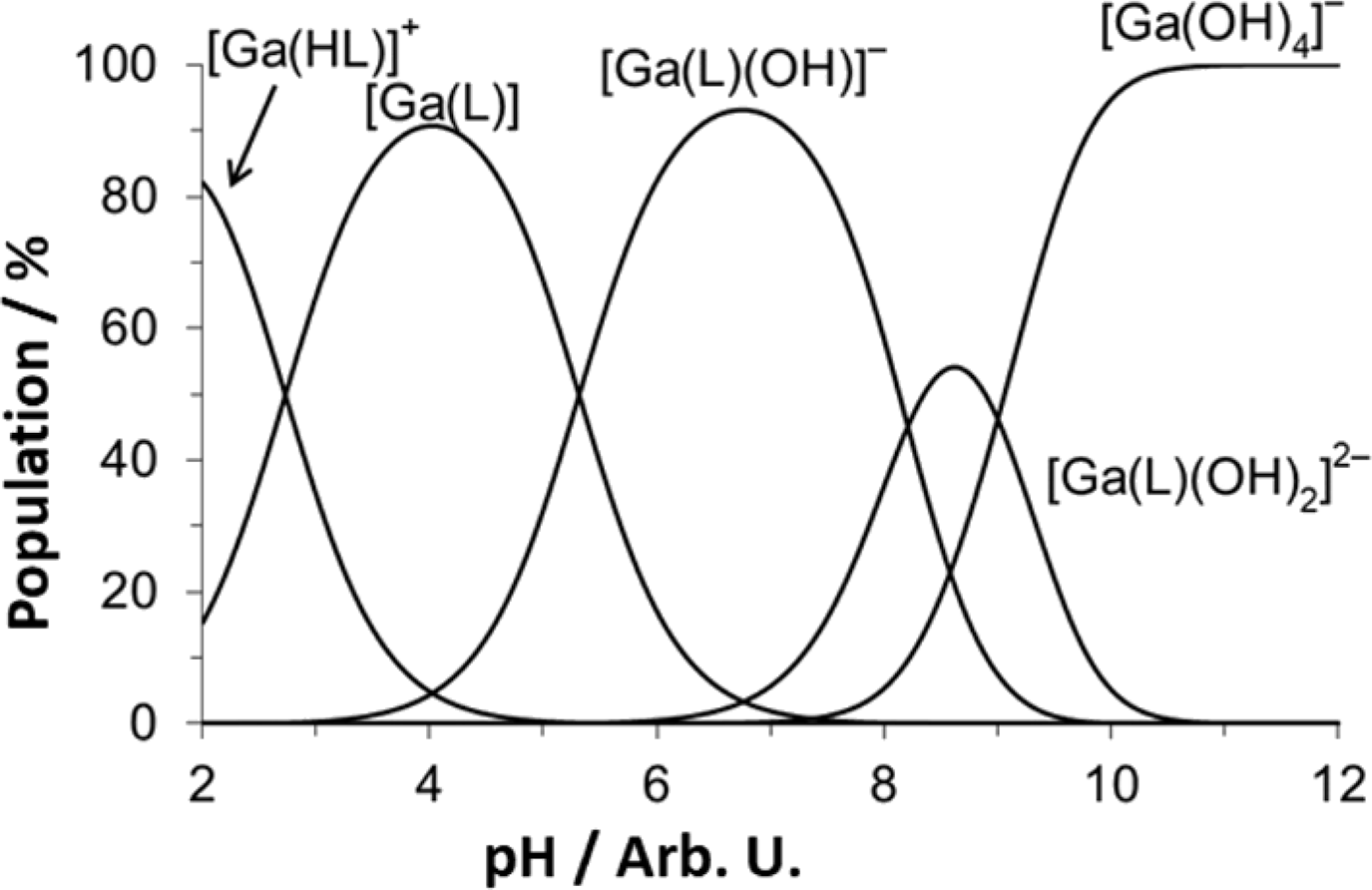
Speciation of Ga^3+^ in solution with **Bn_2_DT3A**. (*T* = 25 °C, *I* = 0.1 M NMe_4_Cl, [**Bn_2_DT3A**] = 4 mM, [Ga^3+^] = 2 mM).

The ligand **Bn_2_DT3A** has a slightly greater affinity for Cu^2+^ than Ga^3+^ (Table 2); the affinity for both ions is greater than that for Zn^2+^. The thermodynamic stability of the [Ga(**Bn_2_DT3A**)] (log *K*[Ga(**Bn_2_DT3A**)] = 18.25, p[Ga(OH)_4_] = 5.78, Table S5) complex is lower than that of the similar systems [Ga(**DTPA**)]^2–^ (log *K*[Ga(**DTPA**)] = 25.11, p[Ga(OH)_4_] = 9.28, Table S5)^40,41^ and [Ga(**NOTA**)] (log *K*[Ga(**NOTA**)] = 29.60, p[Ga(OH)_4_] = 11.82, Table S5).^44^ This is unsurprising in the case of the **NOTA** complex due to the macrocyclic nature of **NOTA**, resulting in improved thermodynamic stability due to pre-organization of the ligand prior to complexation. The difference between [Ga(**Bn_2_DT3A**)] and [Ga(**DTPA**)]^2–^ is more surprising (Figure 2, Table 2, Figure S46, and Table S4)—both ligands likely bind Ga^3+^ in a N_3_O_3_ manner. However, this can be rationalized by considering the ligand basicity; each basic site of **DTPA** is more basic than the equivalent one of **Bn_2_DT3A**. This increased basicity is expected to result in an increase in stability of the formed complex.^44^

**Table 2:** Stability Constants and Dissociation Constants (log β_HLM_) of **Bn_2_DT3A** Complexes^a^

equilibrium	Ga^3+^	Cu^2+^	Zn^2+^
M + L ↔ [M(L)]	18.25	18.9	14.12
[M(HL)] ↔ [M(L)] + H	2.73	2.8	4.16
[M(L)] + H_2_O ↔ [M(L)(OH)] + H	5.32		12.06
[M(L)(OH)] + H_2_O ↔ [M(L)(OH)_2_] + H	8.21		

a Charges are omitted. (*T* = 25 °C, *I* = 0.1 M NMe_4_Cl). The stability constants corresponding to the formation of [Cu(HL)] were determined without ionic strength control.

As has previously been reported for the [Ga(**Dpaa**)(H_2_O)] system (**Dpaa** = 6,6′-{[(carboxymethyl)azanediyl]bis(methylene)}dipicolinic acid, Figure S1), a deprotonation event occurs in the mildly acidic region (p*K_a_* = 5.32, Figure 2).^19,39^ In the case of [Ga(**Dpaa**)(H_2_O)], a coordinated water molecule is the likely site of deprotonation. In the case of [Ga(**Bn_2_DT3A**)], there is no evidence for a coordinated water molecule in the neutral species. As the ligand is fully deprotonated in the [Ga(**Bn_2_DT3A**)] species, this additional deprotonation may be due to coordination of a hydroxide anion to the Ga^3+^ center, replacing one of the donor atoms of the ligand.^49^ A similar exchange has been reported for **PIDAZTA** ligands with Ga^3+^ in which a carboxylate arm is displaced by a hydroxide (p*K_a_* 3.75–4.04).^50^

The Ga-**Bn_2_DT3A** and Ga-**DTPA** distribution diagrams (Figure 2 and Figure S46)^40,41^ show identical species present in solution; however, the pH at which protonated and hydroxide species form differs. The protonated species of Ga-**DTPA** forms at a higher pH (log *K* = 4.06)^40,41^ than that of Ga-**Bn_2_DT3A** (log *K* = 2.73)—this is likely due to the presence of additional carboxylates resulting in easier protonation in acidic solution. In the case of Ga-**Bn_2_DT3A**, this [Ga(HL)] species is likely to be due to protonation of a carboxylate arm, which as a result, is no longer coordinated to the Ga^3+^ center. A similar result is seen in the hydroxide species—the Ga-**DTPA** system forms this product at a higher pH (log *K* = 7.01)^40,41^ than the Ga-**Bn_2_DT3A** system (log *K* = 5.32); this is likely due to the presence of uncoordinated, charged deprotonated carboxylates resulting in a higher resistance to hydroxide attack in alkaline solution. The hydroxide species formed are likely the result of coordination of a hydroxide anion to the Ga^3+^ center with an associated dissociation of one of the ligand coordinating atoms, either an amine or a carboxylate.

The distribution diagram clearly shows that the hydroxide species is the major species at physiological pH and is relevant for *in vitro* and *in vivo* investigations (see below).

### Molecular Modeling

**Figure 3:**
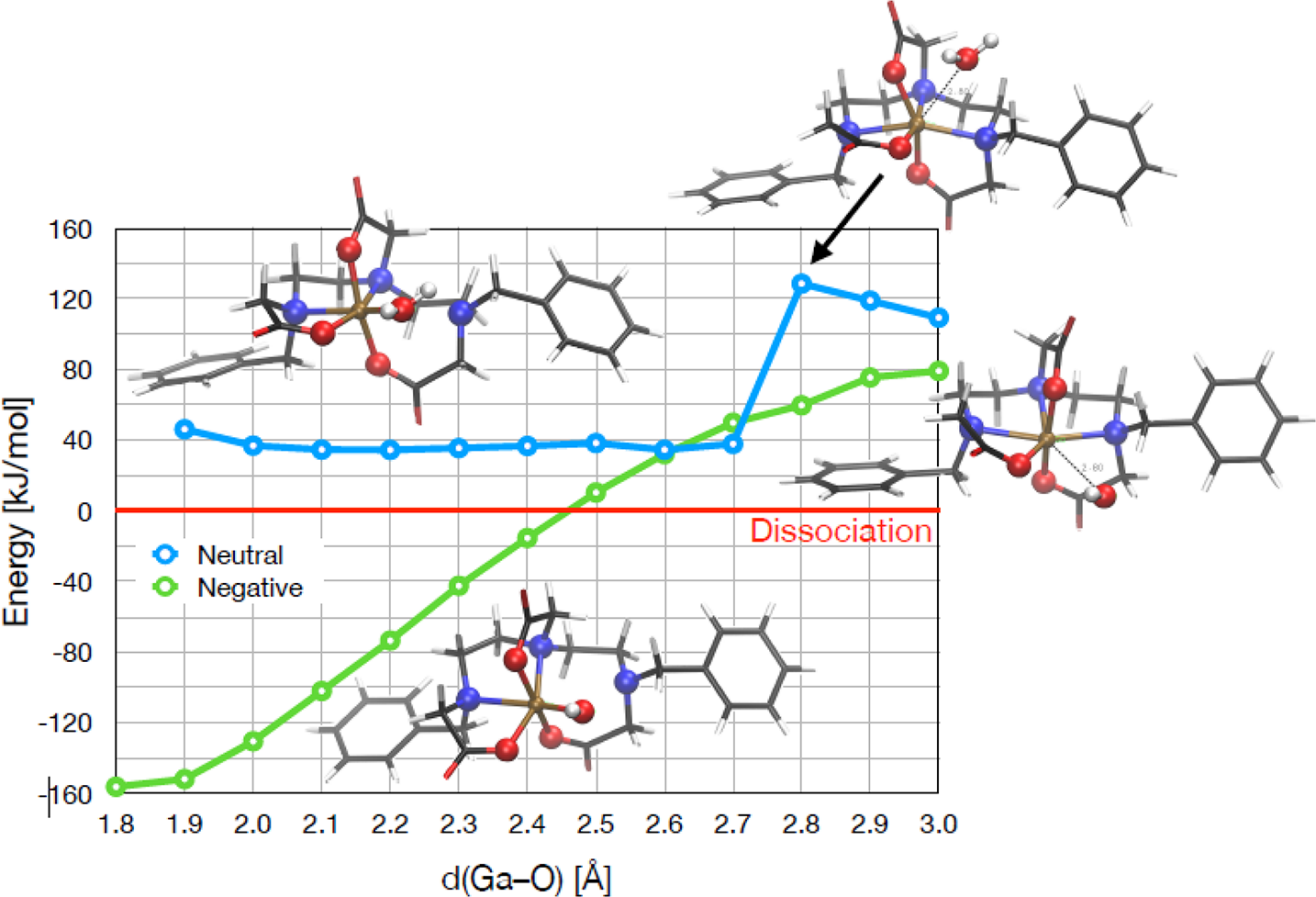
Calculated energy of water molecule (blue) and hydroxide anion (green) interacting with [Ga(**Bn_2_DT3A**)] at various Ga–O distances.

HF-3c calculations show that coordination of a water molecule to the [Ga(**Bn_2_DT3A**)] complex is not thermodynamically favorable (Figure 3). An initial energy barrier of 20 kJ mol^–1^ prevents the water molecule from approaching the Ga^3+^ ion, and, if it were to coordinate to the metal ion, the resulting species is 40 kJ mol^–1^ less stable than the dissociated system. In contrast, a hydroxide ion is shown to be able to approach the Ga^3+^ center, with an overall stabilization of 240 kJ mol^–1^ as it approaches from 3.0 to 1.8 Å. The hydroxide complex is calculated to be 160 kJ mol^–1^ more stable than the dissociated system. The calculations suggest that one of the terminal amine groups is replaced by a hydroxide anion. As the hydroxide coordination can only proceed at sufficient hydroxide anion concentration, the reaction takes place in solution with neutral pH.

### Radiolabeling with ^68^Ga

**Figure 4:**
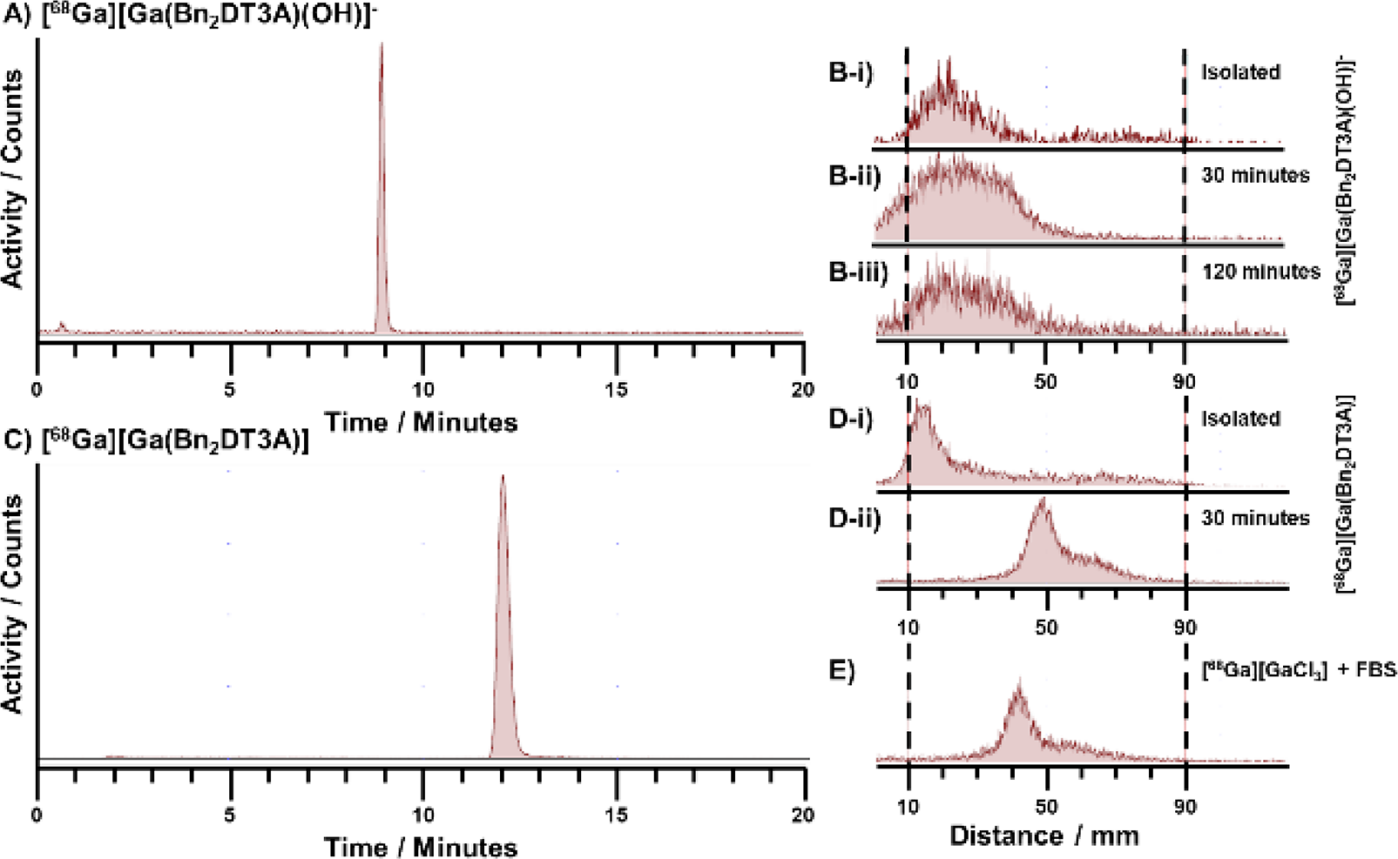
(A) Radio-HPLC of [^68^Ga][Ga(**Bn_2_DT3A**)(OH)]^−^ following semipreparative HPLC purification. (B) Stability of [^68^Ga][Ga(**Bn_2_DT3A**)(OH)]^−^ to FBS assessed by radio-TLC. (i) Isolated species. (ii) After 30 min incubation in FBS. (iii) After 120 min incubation. (C) Radio-HPLC of [^68^Ga][Ga(**Bn_2_DT3A**)] following semipreparative HPLC purification. (D) Stability of [^68^Ga][Ga(**Bn_2_DT3A**)] to FBS assessed by radio-TLC. (i) Isolated species. (ii) After 30 min incubation in FBS. (E) Radio-TLC of [^68^Ga][GaCl_3_] incubated with FBS.

When incubated with ^68^Ga, **Bn_2_DT3A** was found to be capable of achieving high radiochemical yields at both pH 4 and pH 7.4; however, multiple products were formed with pH-dependent abundance. The two radiolabeled products were isolated by semipreparative HPLC (Figure 4) and assessed independently for their stability to fetal bovine serum (FBS, Figure 4). The major product at pH 4, [^68^Ga][Ga(**Bn_2_DT3A**)], was found to be poorly stable to competition by FBS, with none of the complex remaining after 30 min (Figure 4D). In contrast, the major product at pH 7.4, attributed to [^68^Ga][Ga(**Bn_2_DT3A**)(OH)]^−^, was shown to be stable to FBS for over 2 h with no decomplexation seen (Figure 4B and Figure S20). Thus, this radiolabeled product is suitable for further PET applications.

**Figure 5:**
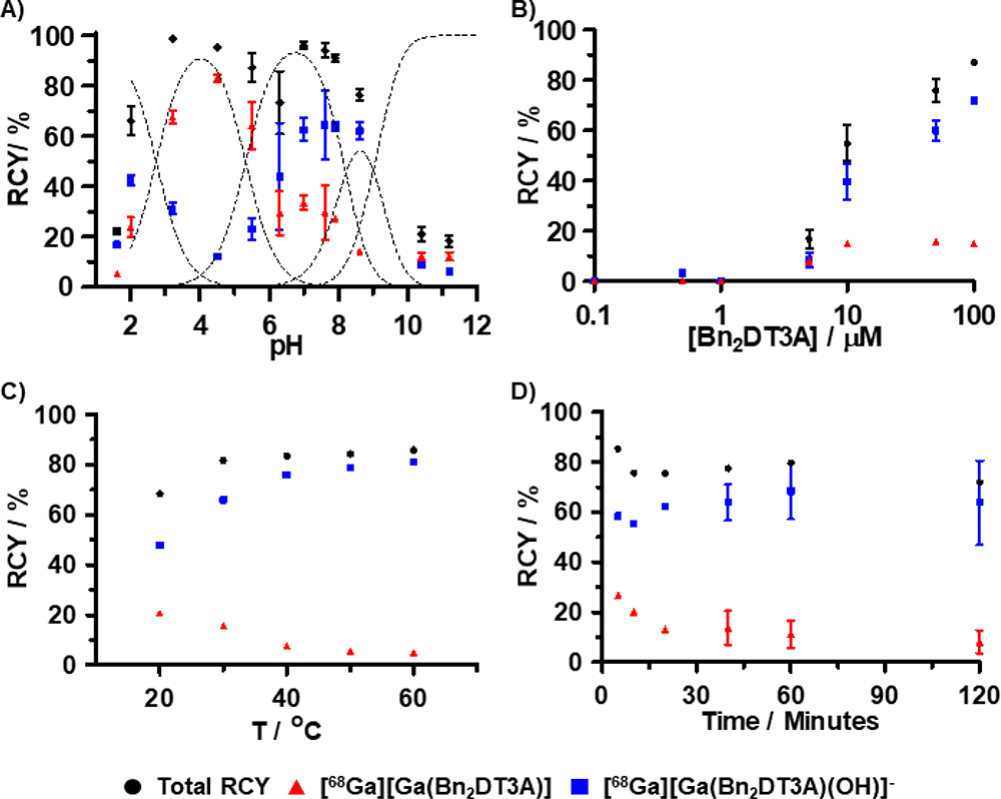
Effect of common reaction parameters on the radiolabeled products of [^68^Ga][GaCl_3_] and **Bn_2_DT3A** as assessed by radio-HPLC. (A) Effect of pH. [**Bn_2_DT3A**] = 100 μM. *T* = 25 °C. *I* = 0.1 M Na_*x*_H_3–*x*_PO_4_. *t* = 15 min. (B) Effect of ligand concentration. *T* = 25 °C. *I* = PBS. p*H* = 7.4. *t* = 15 min. (C) Effect of temperature. [**Bn_2_DT3A**] = 100 μM. *I* = PBS. p*H* = 7.4. *t* = 5 min. (D) Effect of reaction time. [**Bn_2_DT3A**] = 100 μM. *T* = 25 °C. *I* = PBS. p*H* = 7.4.

The effect of pH on the population of the products was further investigated (Figure 5A). At low pH, a negligible amount of the desired hydroxido species was formed; above pH 5, the FBS stable product became more populous. According to the distribution diagram, this stable product corresponds to the species [Ga(**Bn_2_DT3A**)(OH)]^−^ (Figure 5A). This is also supported by its shorter retention time when analyzed by HPLC (Figure 4A), suggesting an increased hydrophilicity due to its charge. The partition coefficient for this species was determined to be log *D*_octanol/PBS_(pH 7.4) = −2.91 +/– 0.07, this fulfills drug development requirements, which is advantageous to future uses as a radiotracer.

The temperature of the radiolabeling reaction and the concentration of the chelator have previously been shown to affect the ratio of diastereomers formed when radiolabeling **HBED** with ^68^Ga;^22,26^ as such, these parameters were also investigated, along with the radiolabeling incubation time. The ligand concentration has a significant impact on the radiochemical yield (RCY); ligand concentrations of at least 100 μM are required to achieve RCYs >90% at pH 7.4 at room temperature in 15 min. The use of a higher ligand concentration in the radiolabeling reaction also promoted the formation of [^68^Ga][Ga(**Bn_2_DT3A**)(OH)]^−^ (Figure 5B).

The temperature of the radiolabeling reaction has a profound impact upon the ratio of the species formed (Figure 5C); elevated temperatures favor the formation of the [Ga(**Bn_2_DT3A**)(OH)]^−^ product with ratios of 20:1 achievable at pH 7.4 and 60 °C after 5 min (Figure S21).

The reaction time has a modest effect on the RCY and the ratio of the species (Figure 5D). The increase in the ratio of species formed with increasing reaction time suggests that there is some slow exchange between the two species; this was not observed when isolating the species by semipreparative HPLC so it may require excess ligand to be present.

The obtained thermodynamic stability constants, HF-3c calculations, and kinetic inertness toward FBS all support the formation of a stable complex in which Ga^3+^ is coordinated by **Bn_2_DT3A** in a five-coordinate manner with the additional coordination site occupied by a hydroxide anion. The formation of this species, [Ga(**Bn_2_DT3A**)(OH)]^−^, occurs only in a significant proportion at pH > 5.

These optimized conditions for the production of [^68^Ga][Ga(**Bn_2_DT3A**)(OH)]^−^ ([*L*] > 100 μM, *T* > 60 °C, *t* > 15 min, pH 7.4) are similar to typical radiolabeling conditions for **DTPA** ([*L*] = 155 μM, *T* = 25 °C, *t* = 20 min, pH = 3.5 or [*L*] = 62 μM, *T* = 80 °C, *t* = 20 min, pH = 3.5)^29,30^ and **CHX-A″-DTPA** ([*L*] = 74 μM, *T* = 95 °C, *t* = 5 min, pH = 3.6–4).^32^ The most noticeable difference is the increased pH of radiolabeling, potentially allowing for the radiolabeling of pH-sensitive motifs with ^68^Ga^3+^ by using **Bn_2_DT3A** as a chelator instead of **DTPA** or **CHX-A″-DTPA**. In terms of stability, less than 60% [^68^Ga][Ga(**DTPA**)] remained intact after 2 h incubation in serum,^31^ and [^68^Ga][Ga(**CHX-A″-DTPA**)] was approximately 85% intact after 2 h^31^—in this comparison, [^68^Ga][Ga(**Bn_2_DT3A**)(OH)]^−^ shows a significant improvement with no decomplexation seen after 2 h incubation with serum. Despite these differences, **Bn_2_DT3A** is still outperformed by the macrocyclic **NOTA**, which is typically radiolabeled at room temperature at much lower ligand concentrations, albeit at acidic pH ([*L*] = 10 μM, *T* = 25 °C, *t* = 10 min, pH = 3.5).^20^

### *In Vivo* Assessment

**Figure 6:**
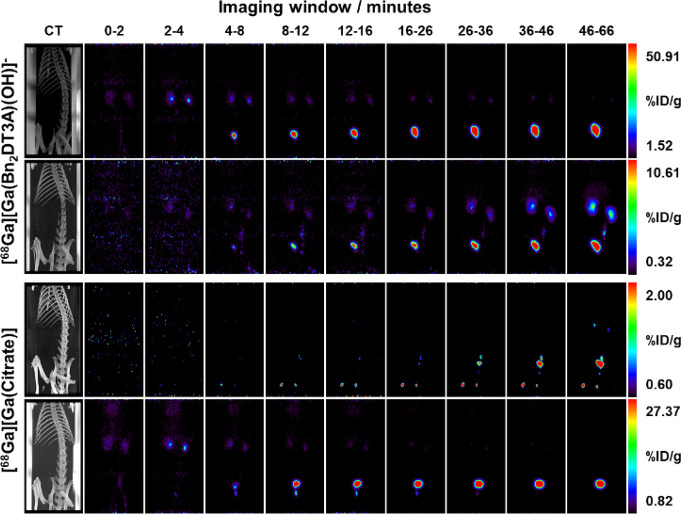
PET scans of healthy Sprague-Dawley rats injected with either [^68^Ga][Ga(**Bn_2_DT3A**)(OH)]^−^ (top two rows) or [^68^Ga][Ga(**Citrate**)]^−^ (bottom two rows) at indicated time points. Subsequent CT scan provided for co-registration of signal.

Following optimization of the radiolabeling conditions, the [^68^Ga][Ga(**Bn_2_DT3A**)(OH)]^−^ complex was investigated *in vivo*. Following semipreparative HPLC purification, the isolated species was reformulated into phosphate buffered saline (PBS) and administered into healthy male Sprague–Dawley rats *via* tail–vein injection. The biodistribution was monitored by sequential PET scans (Figure 6 and Figures S35–S45) followed by a computed tomography (CT) scan to allow for co-registration of the images. The activity rapidly accumulated within the kidneys before passing through the bladder, indicating a renal clearance. No uptake in the liver, lungs, or bones could be observed. When [^68^Ga][Ga(**citrate**)]^−^, a weakly coordinated system in which release of [^68^Ga][Ga^3+^] is expected,^51^ was studied in the same manner, some minor uptake in the lungs and transient localization in the prostate gland of the rats was observed (Figure 6 and Figures S27–S34). Uptake was also observed in the leg joints following injection of [^68^Ga][Ga(**citrate**)]^−^ (Figure 6), which was not observed following injection of [^68^Ga][Ga(**Bn_2_DT3A**)(OH)]^−^. When nonchelated [^68^Ga][Ga^3+^] is injected, high initial uptake is reported in the heart and blood followed by renal clearance with a prolonged heart and blood uptake along with liver and joint uptake.^51,52^ In comparison to these two systems, it is clear that [^68^Ga][Ga(**Bn_2_DT3A**)(OH)]^−^ is rapidly excreted *via* the kidneys with minimal uptake outside of this pathway, suggesting that the ^68^Ga^3+^ ion remains complexed by **Bn_2_DT3A**.

The rapid clearance of [^68^Ga][Ga(**Bn_2_DT3A**)(OH)]^−^ suggests that it will be a good choice of chelate for ^68^Ga PET when conjugated to a targeting moiety. This fast excretion will allow for rapid washout of off-target activity, which will improve the signal-to-noise ratio of the tissues of interest. The biodistribution of a targeted probe incorporating [^68^Ga][Ga(**Bn_2_DT3A**)(OH)]^−^ should be dominated by the targeting motif as no uptake of [^68^Ga][Ga(**Bn_2_DT3A**)(OH)]^−^ in tissues outside of the excretion pathway was observed.

Further development of the system and of bifunctional derivatives could produce a system that can be efficiently labeled at pH 7.4 without heating, resulting in a serum stable product for *in vivo* application.

## Conclusions

A novel hexadentate chelator, **Bn_2_DT3A**, has been prepared and applied to the coordination of Ga^3+^ and to radiochemistry with ^68^Ga.

**Bn_2_DT3A** forms a distorted octahedral *mer-mer* 1:1 complex with Ga^3+^ under acidic conditions with a thermodynamic stability of log *K*[Ga(**Bn_2_DT3A**)] = 18.25. Hydroxide anion coordination occurs with a p*K_a_* of 5.32. HF-3c calculations attribute the species structure to the dissociation of one of the amines and insertion of a hydroxide anion.

**Bn_2_DT3A** is capable of complexing other metal ions, as evidenced by its acceptable Cu^2+^ and Zn^2+^ thermodynamic stability constants. This gives the system a greater versatility, and this is being explored through ^64^Cu^2+^ labeling experiments. While this versatility is often undesired in the design of chelators for radiometals due to the potential for complexation of other metal ions that may be present in the radiolabeling solution, the design of the ligand **Bn_2_DT3A**, with benzyl units that can be substituted to increase or decrease steric hinderance and electronic properties, will allow for optimization of the system to improve selectivity for ^68^Ga^3+^ in the future.

When **Bn_2_DT3A** is radiolabeled with ^68^Ga, two species are formed in a pH-dependent manner. The radiolabeling conditions can be tuned to vary the ratio of these products, and they can be isolated by semipreparative HPLC. The product that is formed above pH 5 and promoted by elevated temperatures and high ligand concentrations was attributed to the deprotonated species [^68^Ga][Ga(**Bn_2_DT3A**)(OH)]^−^. This species was stable to biological competitors for over 2 h in contrast to the neutral species. [^68^Ga][Ga(**Bn_2_DT3A**)(OH)]^−^ was administered to healthy rats and found to have a rapid renal clearance with negligible uptake outside of the clearance pathway.

[Ga(**Bn_2_DT3A**)] shows an increased *in vitro* stability upon hydroxide coordination, which allows it to be used for PET applications. This system is promising for further development of chelators for the complexation of ^68^Ga under mild conditions.

## Supporting Information Available

Experimental details, spectra, speciation diagrams, *in vivo* data, and crystallographic data (CCDC 1864389, 2125953) (PDF)
