# Supplementary material for: Bn2DT3A, a Chelator for 68Ga Positron Emission Tomography: Hydroxide Coordination Increases Biological Stability of [68Ga][Ga(Bn2DT3A)(OH)]−
Source: Inorg Chem. 2022 Oct 17;61(43):17059–67. doi: 10.1021/acs.inorgchem.2c01992 (PMC9627565; doi:10.1021/acs.inorgchem.2c01992)
Supplement: Supplementary file 1 — ic2c01992_si_001.pdf [file ic2c01992_si_001.pdf]

Supplementary Information

Bn_2DT3A, a chelator for ^{68}Ga PET: hydroxide coordination increases biological stability of $[^{68}Ga][Ga(Bn_2DT3A)(OH)]^-$.

Thomas W. Price,[a,b,c] Isaline Renard,[b,c] Timothy J. Prior,[d] Vojtěch Kubíček,[e] David M. Benoit,[f] Stephen J. Archibald,[b,c] Anne-Marie Seymour,[b] Petr Hermann,[e] Graeme J. Stasiuk*[a]

[a] Dr T.W. Price, Dr G.J. Stasiuk

Department of Imaging Chemistry and Biology, School of Biomedical Engineering and Imaging Sciences, King's College London, London, SE1 7EH, UK

E-mail: graeme.stasiuk@kcl.ac.uk

[b] Dr T.W. Price, Dr I. Renard, Prof. S.J. Archibald, Prof. A.-M. Seymour

Department of Biomedical Sciences, University of Hull, Cottingham Road, Hull, HU6 7RX, UK

[c] Dr T.W. Price, Dr I. Renard, Prof. S.J. Archibald

Positron Emission Tomography Research Center, University of Hull, Cottingham Road, Hull, HU6 7RX, UK

[d] Dr T.J. Prior

Chemistry, University of Hull, Cottingham Road, Hull, HU6 7RX, UK

[e] Dr V. Kubíček, Prof. P. Hermann

Department of Inorganic Chemistry, Faculty of Science, Charles University, Hlavova 2030, Prague 2, Czech Republic.

[f] Dr D.M. Benoit

E.A. Milne Centre for Astrophysics, Department of Physics and Mathematics, University of Hull, Cottingham Road, Hull, HU6 7RX, UK.

Figure S1: Structure of chelators discussed in this work

Figure S2: ^{1}H NMR of **2** (400 MHz, D_2O, 298 K)

Figure S3: $^{13}C\{^{1}H\}$ NMR of **2** (100 MHz, D_2O, 298 K)

Figure S4: HMQC of **2** (D_2O, 298K)

Figure S5: HMBC of **2** (D_2O, 298 K)

Figure S6: ^{1}H NMR of **3** (400 MHz, $CDCl_3$, 298 K)

Figure S7: $^{13}C\{^{1}H\}$ NMR of **3** (100 MHz, $CDCl_3$, 298 K)

Figure S8: HMQC of **3** ($CDCl_3$, 298 K)

Figure S9: HMBC of **3** ($CDCl_3$, 298 K)

Figure S10: ^{1}H NMR of **Bn_2DT3A** (400 MHz, D_2O, 298 K)

Figure S11: $^{13}C\{^{1}H\}$ NMR of **Bn_2DT3A** (100 MHz, D_2O, 298 K)

Figure S12: HMQC of **Bn_2DT3A** (D_2O, 298 K)

Figure S13: HMBC of **Bn_2DT3A** (D_2O, 298 K)

Figure S14: ^{1}H NMR of [Ga(**Bn_2DT3A**)(OH)]$^{-}$ (400 MHz, D_2O, pD = 6.8, 298 K)

Figure S15: $^{13}C\{^{1}H\}$ NMR of [Ga(**Bn_2DT3A**)(OH)]$^{-}$ (100 MHz, D_2O, pD = 6.8, 298 K)

Figure S16: HMQC of [Ga(**Bn_2DT3A**)(OH)]$^{-}$ (D_2O, pD = 6.8, 298 K)

Figure S17: HMBC of [Ga(**Bn_2DT3A**)(OH)]$^{-}$ (D_2O, pD = 6.8, 298 K)

Figure S18: ^{1}H NMR spectra of [Ga(**Bn_2DT3A**)] recorded at indicated pD values (400 MHz, D_2O, 298 K)

Figure S19: Radio-HPLC chromatograms of reinjected fractions isolated products A) [^{68}Ga][Ga(**Bn_2DT3A**)], B) [^{68}Ga][Ga(**Bn_2DT3A**)(OH)]$^{-}$

Figure S20: Stability of species formed in radiolabelling of **Bn_2DT3A** with ^{68}Ga following isolation. A) [^{68}Ga][Ga(**Bn_2DT3A**)(OH)]$^{-}$ B) [^{68}Ga][Ga(**Bn_2DT3A**)]. i) isolated species. ii) After 30 min incubation with FBS at 37 °C. iii) after 60 min incubation. iv) 90 min incubation. v) 120 min incubation. vi) [^{68}Ga][$GaCl_3$] incubated with FBS at 37 °C

Figure S21: Effect of temperature on the ratio of products formed during radiolabelling of **Bn_2DT3A** with ^{68}Ga. [**Bn_2DT3A**] = 100 μM, pH = 7.4, *I* = PBS

Figure S22: Distribution diagrams for A) **Bn_2DT3A**, B) Ga(III)-**Bn_2DT3A**, C) Cu(II)-**Bn_2DT3A**, D) Zn(II)-**Bn_2DT3A**. (*T* = 25 °C, *I* = 0.1 M NMe_4Cl, [L] = 4 μM, [M] = 2 μM).

Figure S23: UV-Vis titration of Cu(II)-**Bn_2DT3A** system. Top: UV-Vis spectra variation with pH. Bottom: Difference of absorbance at 650 nm, ΔAbs, used to evaluate results. The line corresponds to the best fit. (*T* = 25 °C, [L] = [M] = 0.01 mM)

Figure S24: ORTEP representation of molecular structure of [Ga(**Bn_2DT3A**)] obtained by single crystal X-ray crystallography (drawn as 30% probability ellipsoids) of a crystal grown at an acidic pH. CCDC Reference: 1864389

Figure S25: Molecular structure of [Ga(**Bn_2DT3A**)] from crystal grown at pH 6.8 determined by X-ray crystallography. Hydrogen atoms have been omitted for clarity. Colors: gallium (pale brown); carbon (gray); nitrogen (blue); oxygen (red).

Figure S26: One view of complex of Ga4 overlayed on complex of Ga1. (Ga4 complex in green; Ga1 complex colored by atom)

Figure S27: Coronal PET and CT scans of male rat injected with [^{68}Ga][Ga(**Citrate**)]$^-$.

Figure S28: PET-CT fused scans of a rat 46–66 min after injection with [^{68}Ga][Ga(**Citrate**)]$^{-}$. A) Transverse projection of upper abdomen. B) Transverse projection of lower abdomen. C) Coronal projection. D) Sagittal projection. Areas of increased uptake are annotated. K = kidneys, B = bladder.

Figure S29: Activity time curves for selected organs following administration of [^{68}Ga][Ga(**Citrate**)]$^{-}$.

Figure S30: Activity-time curves for additional regions of increased uptake following administration of [^{68}Ga][Ga(**Citrate**)]$^{-}$.

Figure S31: Coronal PET and CT scans of male rat injected with [^{68}Ga][Ga(**Citrate**)]$^{-}$.

Figure S32: Fused PET-CT scans of a rat after injection with [^{68}Ga][Ga(**Citrate**)]$^{-}$. A) Transverse projection of upper abdomen 2–4 min after injection. B) Transverse projection of lower abdomen 12–16 min post injection. C) Coronal projection 12–16 min post injection. D) Sagittal projection 12–16 min post injection. Areas of increased uptake are annotated. K = kidneys, B = bladder.

Figure S33: Activity time curves for selected organs following administration of [^{68}Ga][Ga(**Citrate**)]$^{-}$.

Figure S34: Activity-time curves for additional regions of increased uptake following administration of [^{68}Ga][Ga(**Citrate**)]$^{-}$.

Figure S35: Coronal PET and CT scans of male rat injected with [^{68}Ga][Ga(**Bn$_2$DT3A**)(OH)]$^{-}$

Figure S36: Fusion PET-CT scans of rat injected with [^{68}Ga][Ga(**Bn$_2$DT3A**)(OH)]$^{-}$ 18–23 min post injection. A) Transverse projection. B) Coronal projection. C) Sagittal projection. Areas of increased uptake are annotated. K = kidneys.

Figure S37: Activity time curves for selected organs following administration of [^{68}Ga][Ga(**Bn$_2$DT3A**)(OH)]$^{-}$.

Figure S38: Activity-time curves for additional regions of increased uptake following administration of [^{68}Ga][Ga(**Bn$_2$DT3A**)(OH)]$^{-}$.

Figure S39: Coronal PET and CT scans of male rat injected with [^{68}Ga][Ga(**Bn$_2$DT3A**)(OH)]$^{-}$

.

Figure S40: Fused PET-CT scans of a rat 46–66 min post injection with [^{68}Ga][Ga(**Bn$_2$DT3A**)(OH)]$^{-}$. A) Transverse projection of upper abdomen. B) Transverse projection of lower abdomen. C) Coronal projection. D) Sagittal projection. Areas of increased uptake are annotated. K = kidneys, B = bladder.

Figure S41: Activity time curves for selected organs following administration of $[^{68}Ga][Ga(\mathbf{Bn_2DT3A})(OH)]^{-}$.

Figure S42: Activity-time curves for additional regions of increased uptake following administration of $[^{68}Ga][Ga(\mathbf{Bn_2DT3A})(OH)]^{-}$.

Figure S43: Coronal PET and CT scans of male rat injected with $[^{68}Ga][Ga(\mathbf{Bn_2DT3A})(OH)]^{-}$.

Figure S44: Fused PET-CT scans of a rat 36–46 min post injection with $[^{68}Ga][Ga(\mathbf{Bn_2DT3A})(OH)]^{-}$. A) Transverse projection of upper abdomen. B) Transverse projection of lower abdomen. C) Coronal projection. D) Sagittal projection. Areas of increased uptake are annotated. K = kidneys, B = bladder.

Figure S45: Activity time curves for selected organs following administration of $[^{68}Ga][Ga(\mathbf{Bn_2DT3A})(OH)]^{-}$.

Figure S46: Speciation of Ga^{3+} in solution with DTPA. (T = 25 °C, I = 0.1 M KNO_3, [DTPA] = 4 mM, $[Ga^{3+}]$ = 2 mM).[1]

Table S1: Overall protonation constants of ligands and stability constants (log*β*) of complexes. (*T* = 25 °C, *I* = 0.1 M NMe_4Cl). Charges are omitted. [a]The stability constants corresponding to the formation of [Cu(HL)] were determined without ionic strength control.

Table S2: Crystal data and structure refinement for [Ga(**Bn_2DT3A**)] for crystal grown at acidic pH. CCDC Reference: 1864389

Table S3: Crystal data and structure refinement for [Ga(**Bn_2DT3A**)] for crystal grown at pH 6.8. CCDC Reference: 2125953

Table S4: Protonation constants of DTPA and stability constants of its Ga(III) complexes (*I* = 0.1 M KNO_3).[1]

Table S5: pGa (– logc_{Ga3+}) and p[Ga$(OH)_4$] (– log$c_{[Ga(OH)4]-}$) values for selected chelators. The pGa and p[Ga$(OH)_4$] values were calculated for pH 7.4, c_{Ga} = 1.0 μM, c_L = 10 μM.

1. General

MATERIALS & METHODS

Unless otherwise stated, all chemicals were purchased from Sigma Aldrich (Dorset, UK), and all solvents were purchased from VWR (Leicestershire, UK). All commercially available starting materials were used without further purification. Unless otherwise stated, all reactions that required anhydrous conditions or involved moisture sensitive compounds were performed under an atmosphere of dry argon.

NMR spectra were recorded on a JEOL ECP 400 MHz/JEOL Lambda 400 MHz spectrometer using the residual protic solvent signal as an internal reference. Chemical shifts (δ) are reported in parts per million (ppm). Coupling constants (J) were measured in hertz (Hz) and NMR multiplicity are abbreviated as follows: s = singlet, d = doublet, t = triplet, q = quartet, quin = quintet, m = multiplet, br = broad. ESI Mass spectra were recorded on Advion MS SOP electrospray ionisation spectrometer. High-resolution mass spectra were collected by the University of Hull Mass Spectrometry service on a maXis impact instrument. Elemental analysis was performed by the University of Hull Elemental analysis service. UV-Vis absorption spectra were recorded on a Thermo Scientific

Evolution 300 UV-Vis spectrometer using quartz cuvettes. p*H* measurements were carried out using a Jenway model 3520 pH/mV/temperature meter with a three-point calibration.[2]

Crystals of a suitable quality for analysis were prepared by slow evaporation of an aqueous solution of the complex. Single-crystal X-ray diffraction data were collected from a crystal at 100 K using a STOE STADI VARI 200K Pilatus diffractometer operating with Cu-radiation (Xenocs, Cu-Genix 3D HF Microfocus source). The structure was solved using dual-space methods within SHELXT[3] and refined using SHELXL.[4]

TLC was performed on Kieselgel 60 F_{254} plates (Merck, Kenilworth, USA) and the spots were visualized in UV (254 and 366 nm) or I_2 vapor. Column chromatography was carried out with silica gel (0.040 – 0.063 mm, Merck, Kenilworth, USA). Analytical HPLC was performed using an Agilent Zorbax Eclipse XDB-C18 column (4.6 × 150 mm with 4.6 × 12.5 mm guard column) and the indicated solvents and gradient. Semi-preparative HPLC was performed using an Agilent Zorbax Eclipse XDB-C18 column (9.4 × 250 mm). Flow Rate: 1 ml min^{-1}. Solvent A: MeOH. Solvent B: Water + 0.1% trifluoroacetic acid. Gradient, ([Time / min] Solvent A: Solvent B): [0] 5:95, [3] 5:95, [12] 60:40, [13], 95:5, [16] 95:5, [17] 5:95, [20] 5:95

Potentiometry

Potentiometry was carried out according to previously published procedures.[5,6] Protonation and stability constants were determined in 0.1 M $(NMe_4)Cl$ at 25.0 °C with pK_w = 13.81. Protonation constants ([L] = 0.004 M) were determined from data obtained in pH range 1.6–12.1 (~40 points per titration and three parallel titrations) with electrode calibrated by acid–base titration in extended pH ranges (1.7–12.2 for each titration set). Complex stability constants ([L] = [M] = 0.004 M) were determined from data obtained in pH range 1.5–12.1, 50 data points per titration, three parallel titrations. Solutions of Ga^{3+}-**Bn$_2$DT3A** complex had to be equilibrated for 30 min before the titration start to reach the full complexation at pH 1.6. Due to the complete formation of Ga^{3+}-**Bn$_2$DT3A** complex at acidic pH, the stability was assessed by competition with the tetrahydroxidogallate species, $[Ga(OH)_4]^-$, which forms in the alkaline region. The stability constants of the Ga^{3+}-hydroxido species were adopted from literature as follows: logβ(Ga(OH)) = 11.21, logβ($Ga(OH)_2$) = 21.72, logβ($Ga(OH)_3$) = 31.13, logβ($Ga(OH)_4$) = 38.64 (data corresponds to equilibrium: $Ga^{3+} + n\ H_2O \rightarrow [Ga(OH)_n]^{3-n} + n\ H^+$).[7] UV-VIS spectra were recorded on spectrophotometer Specord 50 Plus (Analytik Jena AG). Temperature was maintained by Peltier block. UV-VIS titration of Cu^{2+}-**Bn$_2$DT3A** system ([L] = 0.004 M, [M] = 0.002 M, Figure S23) was performed at pH range 0.0–1.8 without ionic strength control, pH was calculated from added amount of HCl. The titration and UV-VIS data were treated

simultaneously with OPIUM program package.[8,9] Calculated constants are concentration constants defined as $\beta_{hl} = [H_hL_l]/[H]^h \cdot [L]^l$ or $\beta_{hlm} = [H_hL_lM_m]/[H]^h \cdot [L]^l \cdot [M]^m$ and standard deviations are given directly by the program (Tables S1). The calculated overall stability constants are given in SI (Table S1). pGa and p[Ga(OH)$_4$]values (Table S5) were calculated as the negative logarithm of free gallium(III) aqua ion and tetrahydroxidogallate concentration, respectively. The pGa values were calculated for pH 7.4, c_{Ga} = 1.0 μM, c_L = 10 μM.

Radiolabelling

^{68}Ga was obtained from either an IGG100 (Eckert & Ziegler, Berlin, Germany) or an iThemba (iThemba, Somerset West, South Africa) ^{68}Ge/^{68}Ga generator by elution with aq. 0.6 M HCl (4 mL). This eluent was diluted with water (20 mL) and the solution passed through a Strata-X-C 33 μm Cation Mixed-mode polymeric support under vacuum. The activity was trapped on the cartridge. The activity was liberated using 98% acetone-2% aq. 0.1 M HCl solution (1 mL).

Aliquots (~30 MBq) of this solution were dried under a stream of inert gas at 90 °C and allowed to cool before use. Ligand solution (1 mL) was preheated and added to the dried

^{68}Ga and shaken at the reaction temperature. Aliquots (5 μL and 20 μL) were taken for analysis by TLC and HPLC, respectively.

TLC analysis was performed on Kieselgel 60 F_{254} plates with an eluent of aq. 0.1 M citric acid. Isolation of the radiolabeled product was achieved by semi-preparative HPLC.

Assessment of stability to foetal bovine serum

100 μL of isolated [^{68}Ga][Ga(**Bn$_2$DT3A**)] or [^{68}Ga][Ga(**Bn$_2$DT3A**)(OH)]$^-$ was added to 1.5 mL of fetal bovine serum and incubated at 37 °C. Aliquots were taken at 30 min intervals for TLC analysis.

Measurement of octanol-PBS partition coefficient

[^{68}Ga][Ga(**Bn$_2$DT3A**)(OH)]$^-$ in PBS (10 μL) was added to PBS (90 μL) and octanol (100 μL). The solution was shaken for 15 min at room temperature before being separated by centrifugation at 15 krpm for 10 min. The layers were separated and their activity measured with a gamma counter.

***In vivo* investigation of [^{68}Ga][Ga(Bn$_2$DT3A)(OH)]$^-$**

Bn$_2$DT3A (500 μM) in 0.1 M phosphate buffer (1 mL, pH 7.8) was added to dried [^{68}Ga]$GaCl_3$ and the solution shaken at room temperature for 15 min.

The solution was purified by semi-preparative HPLC and the isolated fraction (200 μL) diluted with water (2 mL) and trapped on an Oasis Hydrophilic-Lipophilic Balanced cartridge. The cartridge was washed with water (2 mL) and dried with argon. The activity was eluted with ethanol (500 μL) and dried at 99 °C under argon for 10 min. The dried activity was resuspended in PBS (200 μL) and passed through a sterile filter before use.

To dried [^{68}Ga]GaCl_3 was added sodium citrate in water (1 mM, 1 mL). The solution was shaken for 15 min at room temperature. The pH was adjusted to 7 with aq. sodium hydroxide and the solution passed through a sterile filter before use.

A male Sprague Dawley rat (500 g) was induced using isoflurane (5%) and anesthatised state maintained with isoflurane (3%) in oxygen (1 L min^{-1}). The solution of [^{68}Ga][Ga(**Bn_2DT3A**)(OH)]$^-$ in PBS or [^{68}Ga][Ga(**Citrate**)]$^-$ in water was administered *via* tail vein injection.

Whole body PET data acquisition (2 or 3 bed positions, 23- or 66-min dynamic scan) was synchronised with radiotracer *i.v.* injection (2–4 MBq, 200 μL), a CT scan (40 kV, 140 μA, 360 projections, 8 shots) was acquired following each PET scan to show anatomical co-registration; temperature and respiration were monitored throughout the scan. PET-CT imaging data were acquired on a Sedecal SuperArgus 2R PET scanner. PET data were

reconstructed using 3D Ordered Subset Expectation Maximisation (OSEM3D) algorithm with 16 subsets and 2 iterations and corrections for randoms, scatter and attenuation. Images were normalised using the injected dose and animal weight to give Standardised Uptake Values (SUV). Data was analysed using AMIDE software.

Synthesis of N_1-benzyl-N_2-(2-(benzylamino)ethyl)ethane-1,2-diamine (2)[10]

To diethylenetriamine (**1**, 4.78 g, 5.0 mL, 46.3 mmol, 1 eq) in EtOH (50 mL) was added benzaldehyde (10.28 g, 9.9 mL, 96.8 mmol, 2 equiv.). The solution was heated to reflux for 4 h. After cooling to 0 °C, sodium borohydride (12.26 g, 324.2 mmol, 7 equiv.) was added and the solution was stirred at room temperature overnight. The solution was concentrated giving a yellow/white foamy solid. Aq. 1 M NaOH (250 mL) was added and extracted with chloroform (3 x 250 mL). The combined organic extracts were washed with aq. 1 M NaOH (200 mL). After drying with anhydrous $MgSO_4$, the organic layers were concentrated to approximately 300 mL. Conc. aq. HCl (12.5 mL) was added with a large amount of white precipitate forming. This was allowed to cool before being collected on a sinter and washed with chloroform and diethyl ether. The white solid was dried on a high vacuum to yield a white solid (14.17 g, 29.3 mmol, 63%). ^{1}H NMR (400 MHz, D_2O, 298 K), δ: 7.44-7.37 (m, 10 H, NCH_2***Ph***), 4.21 (br s, 4 H, NC$\boldsymbol{H_2}$Ph), 3.38-3.18 (m, 8 H,

NC$\boldsymbol{H_2}$C$\boldsymbol{H_2}$N). $^{13}C\{^1H\}$ NMR (100 MHz, D_2O, 298 K), δ: 130.11 (NCH_2***Ph***), 129.89 (NCH_2***Ph***), 129.79 (NCH_2***Ph***), 129.34 (NCH_2***Ph***), 51.47 (N***C***H_2Ph), 43.61 (N***C***H_2***C***H_2N). MS (ESI) *m/z*: 284.5 $[M+H]^+$

Synthesis of di-*t*-butyl 2,2′-((((2-(*t*-butoxy)-2-oxoethyl)azanediyl)bis(ethane-2,1-diyl))bis(benzylazanediyl))diacetate (3)[11]

To **2** (6.06 g, 15.4 mmol, 1 equiv.) in acetonitrile (200 mL) was added sodium carbonate (13.08 g, 123.4 mmol, 8 equiv.). *Tert*-butyl bromoacetate (9.18 g, 7.0 mL, 47.1 mmol, 3.05 equiv.) was added slowly. The suspension was heated to 60 °C for 16 h. The reaction was quenched by addition of water (1 L) and extracted with dichloromethane (3 x 1 L). The combined organic layers were dried with anhydrous $MgSO_4$ and concentrated to yield a yellow oil. Purification by column chromatography (silica, dichloromethane/triethylamine, 1%) yielded a yellow oil (4.56 g, 7.3 mmol, 47%). ^1H NMR (400 MHz, $CDCl_3$, 298 K), δ: 7.39-7.28 (m, 8 H, NCH_2***Ph***), 7.23 (t, 2 H, NCH_2***Ph***, $^3J_{HH}$ = 7.1 Hz), 3.78 (s, 4 H, NC$\boldsymbol{H_2}$Ph), 3.32 (s, 2 H, NC$\boldsymbol{H_2}$$CO_2$^{t}Bu), 3.23 (s, 4 H, NC$\boldsymbol{H_2}$$CO_2$^{t}Bu), 2.76 (br s, 8 H, NC$\boldsymbol{H_2}$C$\boldsymbol{H_2}$N), 1.46 (s, 18 H, $CO_2C(C\boldsymbol{H_3})_3$), 1.42 (s, 9 H, $CO_2C(C\boldsymbol{H_3})_3$). $^{13}C\{^1H\}$ NMR (100 MHz, $CDCl_3$, 298 K), δ: 170.97 (***C***O_2^{t}Bu), 170.85 (***C***O_2^{t}Bu), 139.08 (NCH_2***Ph***), 128.96 (NCH_2***Ph***), 128.19 (NCH_2***Ph***), 126.97 (NCH_2***Ph***), 80.70 (CO_2***C***$(CH_3)_3$), 80.65 (CO_2***C***$(CH_3)_3$), 58.30

(NCH$_2$Ph), 55.94 (NCH$_2$CO$_2$^{t}Bu), 55.11 (NCH$_2$CO$_2$^{t}Bu), 52.48 (NCH$_2$$CH_2$N), 51.92 (N$CH_2$$CH_2$N), 28.19 (CO$_2$C($CH_3$)$_3$), 28.14 (CO$_2$C($CH_3$)$_3$). MS (ESI), *m/z*: 626.2 $[M+H]^+$. HRMS (ESI), m/z: 626.4168 (Calculated for $[M+H]^+$,$C_{36}H_{56}N_3O_6$: 626.4164)

Synthesis of 2,2′-((((carboxymethyl)azanediyl)bis(ethane-2,1-diyl))bis(benzylazanediyl))diacetic acid (Bn_2DT3A)

To **3** (2.710 g, 4.33 mmol, 1 equiv.) in dichloromethane (10 mL) at 0 °C was added trifluoroacetic acid (10 mL). The solution was stirred at 0 °C for 2 h. The solution was allowed to warm to room temperature and stirred for 24 h. The solution was concentrated and triturated with dichloromethane and ether to yield a white solid (2.00 g, 3.43 mmol, 79%). ^{1}H NMR (400 MHz, D_2O, 298 K), δ: 7.43 (br s, 10 H, NCH$_2$***Ph***), 4.34 (s, 4 H, NC***H***$_2$Ph), 3.90 (s, 4 H, NC***H***$_2$CO$_2$H), 3.18 (t, 4 H, NC***H***$_2$C***H***$_2$N, $^3J_{HH}$ = 5.9 Hz), 3.12 (s, 2 H, NC***H***$_2$CO$_2$H), 2.79 (t, 4 H, NC***H***$_2$C***H***$_2$N, $^3J_{HH}$ = 5.9 Hz). ^{13}C{^{1}H} NMR (100 MHz, D_2O, 298 K), δ: 175.03 (CO$_2$H), 168.86 (CO$_2$H), 131.20 (NCH$_2$***Ph***), 130.52 (NCH$_2$***Ph***), 129.48 (NCH$_2$***Ph***), 128.41 (NCH$_2$***Ph***), 59.44 (NCH$_2$Ph), 54.19 (NCH$_2$CO$_2$H), 51.21 (NCH$_2$$CH_2$N), 48.97 (N$CH_2$$CH_2$N). MS (ESI) *m/z*: 458.8 $[M+H]^+$. HRMS (ESI), *m/z*: 458.2289 (Calculated for $[M+H]^+$, $C_{22}H_{32}N_3O_6$: 458.2286). Elemental Analysis Found, %: C, 53.91,

H, 5.44, N, 7.22 (Calculated for $(H_3Bn_2DT3A)(TFA)_{1.1}$, $C_{26.2}H_{32.1}F_{3.3}N_3O_{8.2}$, %: C, 53.98, H, 5.55, N, 7.21).

Ga^{3+} complexation by Bn_2DT3A

To **Bn_2DT3A** (99 mg, 220 μmol) in water (10 mL) was added $GaCl_3$ (42 mg, 240 μmol). The pH was adjusted to 4 with NaOH and the solution heated to reflux for 16 h. The solution was concentrated to yield a white/yellow solid (187 mg) which was used without further purification. ^{1}H NMR (400 MHz, D_2O, pD = 6.8, 298 K), δ: 7.53-7.37 (m, 10 H, $NCH_2\boldsymbol{Ph}$), 4.27 (d, 1 H, $NC\boldsymbol{H_2}Ph$, $^2J_{HH}$ = 17 Hz), 4.19 (d, 1 H, $NC\boldsymbol{H_2}Ph$, $^2J_{HH}$ = 13 Hz), 4.05 (d, 1 H, $NC\boldsymbol{H_2}CO_2^-$, $^2J_{HH}$ = 17 Hz), 3.96 (d, 1 H, $NC\boldsymbol{H_2}Ph$, $^2J_{HH}$ = 13 Hz), 3.91-3.56 (m, 5 H, $NC\boldsymbol{H_2}Ph$, $NC\boldsymbol{H_2}CO_2^-$, $NC\boldsymbol{H_2}C\boldsymbol{H_2}N$), 3.52-3.29 (m, 4 H, $NC\boldsymbol{H_2}C\boldsymbol{H_2}N$, $NC\boldsymbol{H_2}CO_2^-$), 3.17 (t, 2 H, $NC\boldsymbol{H_2}C\boldsymbol{H_2}N$, $^2J_{HH}$ = 16 Hz), 3.08 (t, 2 H, $NC\boldsymbol{H_2}C\boldsymbol{H_2}N$, $^2J_{HH}$ = 15 Hz), 2.67 (m, 1 H, $NC\boldsymbol{H_2}C\boldsymbol{H_2}N$). $^{13}C\{^1H\}$ NMR (100 MHz, D_2O, pD = 6.8, 298 K), δ: 175.14 ($\boldsymbol{C}O_2^-$), 174.94 ($\boldsymbol{C}O_2^-$), 132.74 ($NCH_2\boldsymbol{Ph}$), 131.64 ($NCH_2\boldsymbol{Ph}$), 129.41 ($NCH_2\boldsymbol{Ph}$), 128.88 ($NCH_2\boldsymbol{Ph}$), 57.27 ($N\boldsymbol{C}H_2Ph$), 56.96 ($N\boldsymbol{C}H_2CO_2^-$), 56.60 ($N\boldsymbol{C}H_2CO_2^-$), 54.84 ($N\boldsymbol{C}H_2\boldsymbol{C}H_2N$), 49.50 ($N\boldsymbol{C}H_2\boldsymbol{C}H_2N$). HRMS (ESI), *m/z*: 524.1734 (Calculated for $[^{69}Ga][M+H]^+$, $C_{24}H_{39}GaN_3O_6$: 524.1307).

Computational studies

HF-3c calculations[12] were performed using ORCA v4.0.1.[13] Each energy profile was obtained by fully optimizing all coordinates of the Ga^{3+} complex using default convergence criteria, while constraining the Ga–O distance of the approaching ligand (OH_2 or OH^-) along the energy path. To account for the effect of solvation on the complex, we use a Conductor-like Polarizable Continuum Model (C-PCM) solvation model parametrised for water using a Conductor-like Screening Solvation Model (COSMO) epsilon function. We estimate the dissociation energy of the complex by computing the total energy for a ligand held 6.5 Å away from the Ga^{3+} centre.

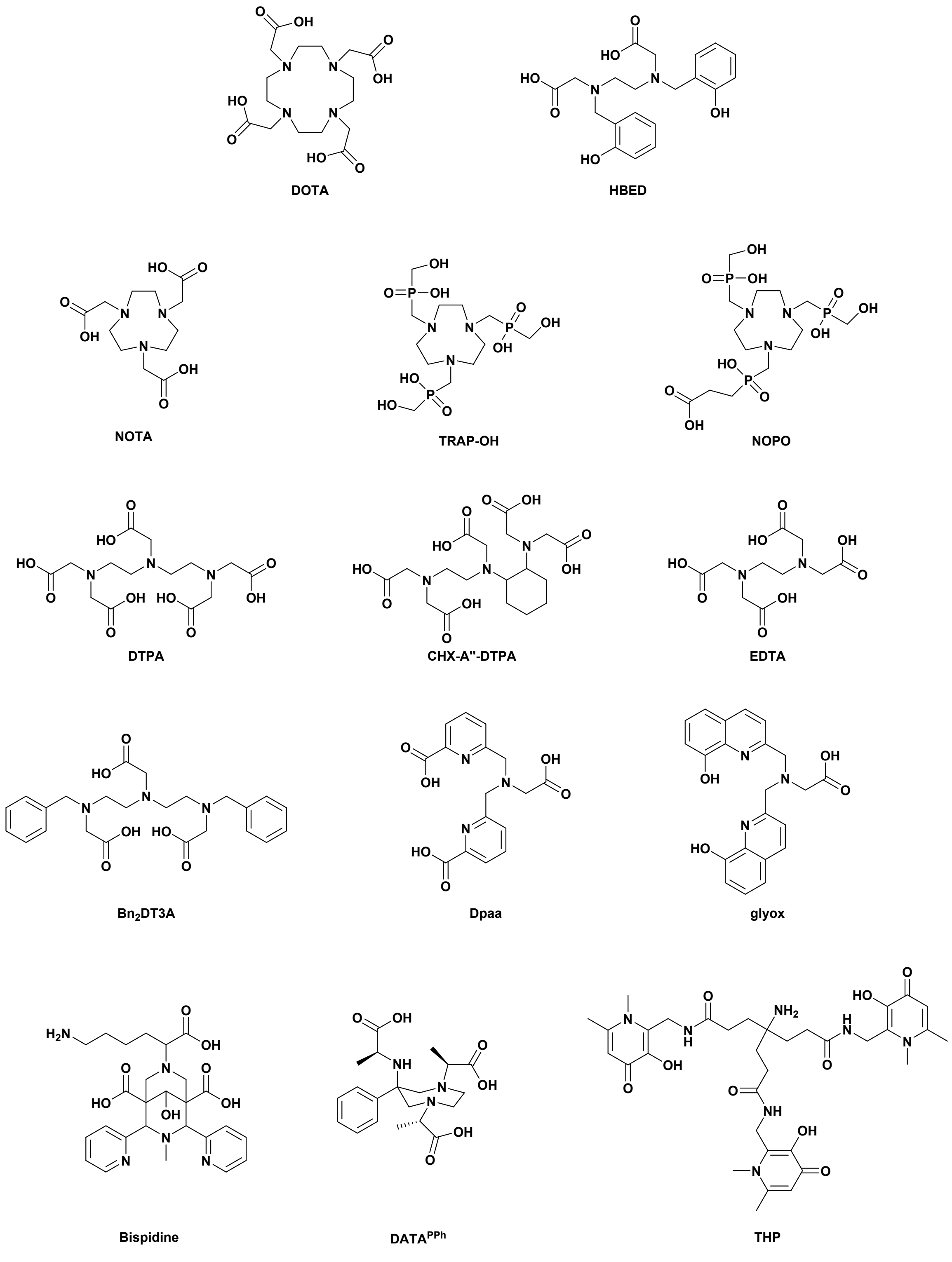

Figure S1: Structure of chelators discussed in this work

2. NMR Data

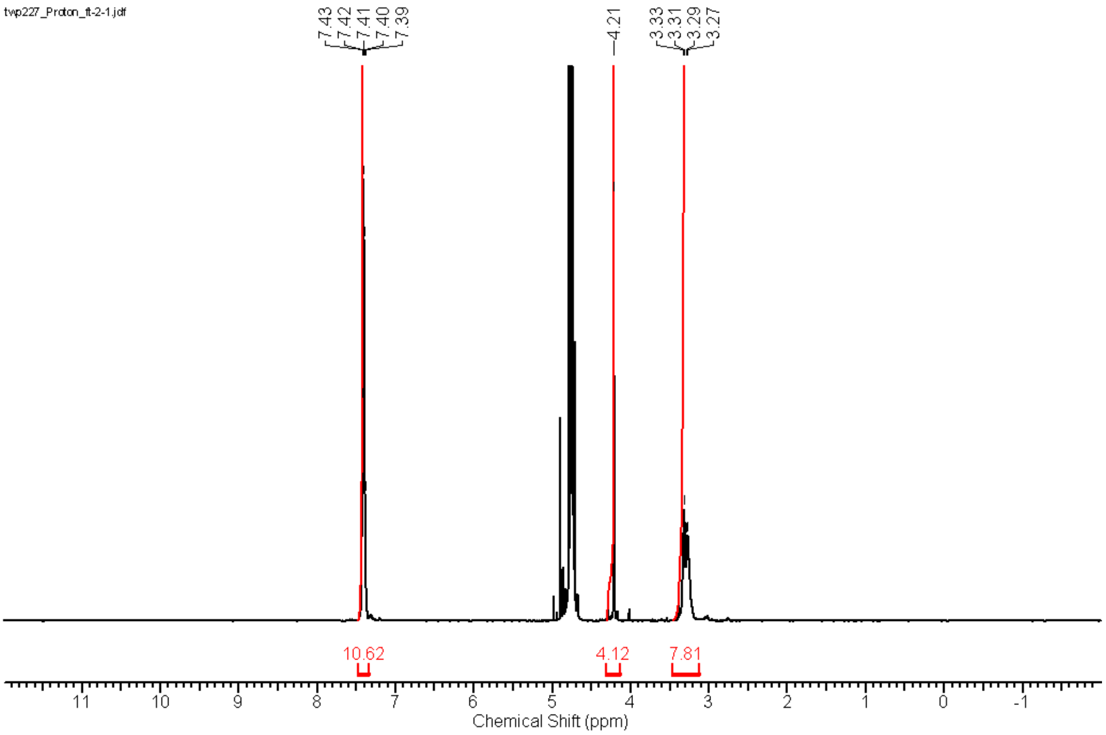

Figure S2: ^{1}H NMR of **2** (400 MHz, D_2O, 298 K)

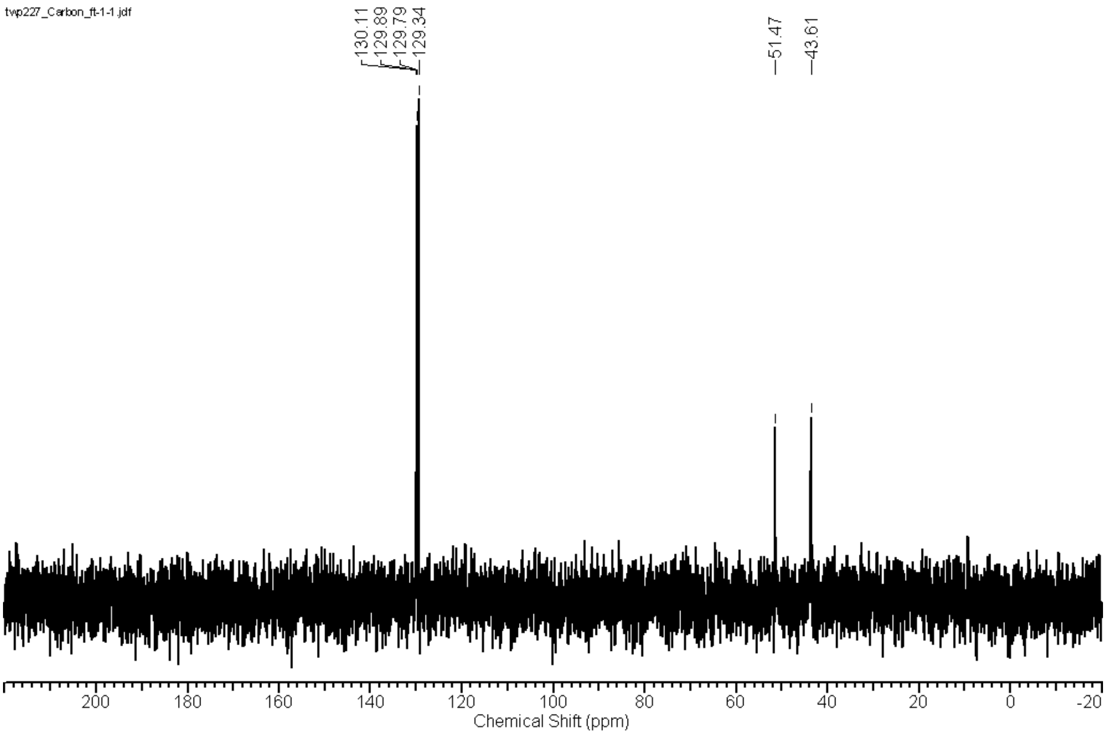

Figure S3: $^{13}C\{^1H\}$ NMR of **2** (100 MHz, D_2O, 298 K)

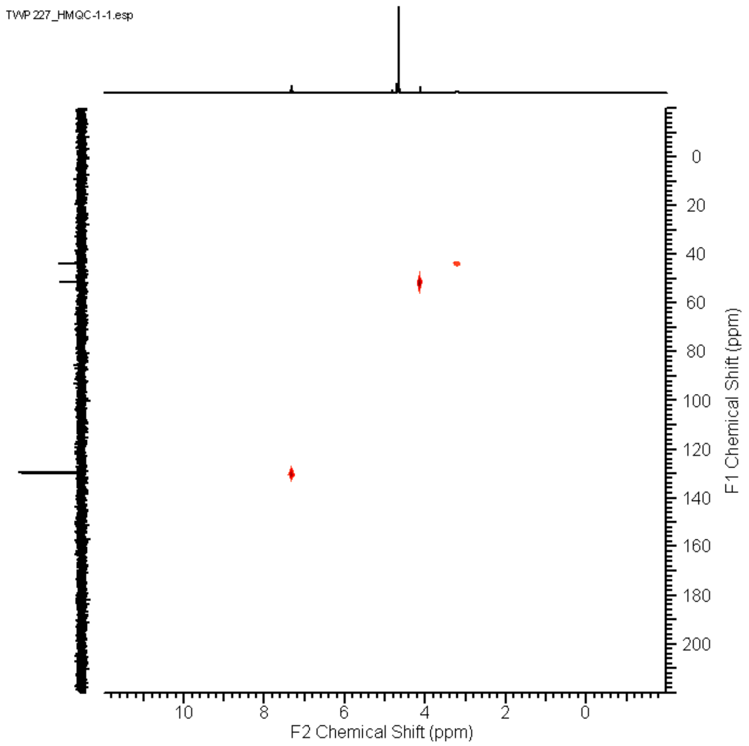

Figure S4: HMQC of **2** (D_2O, 298K)

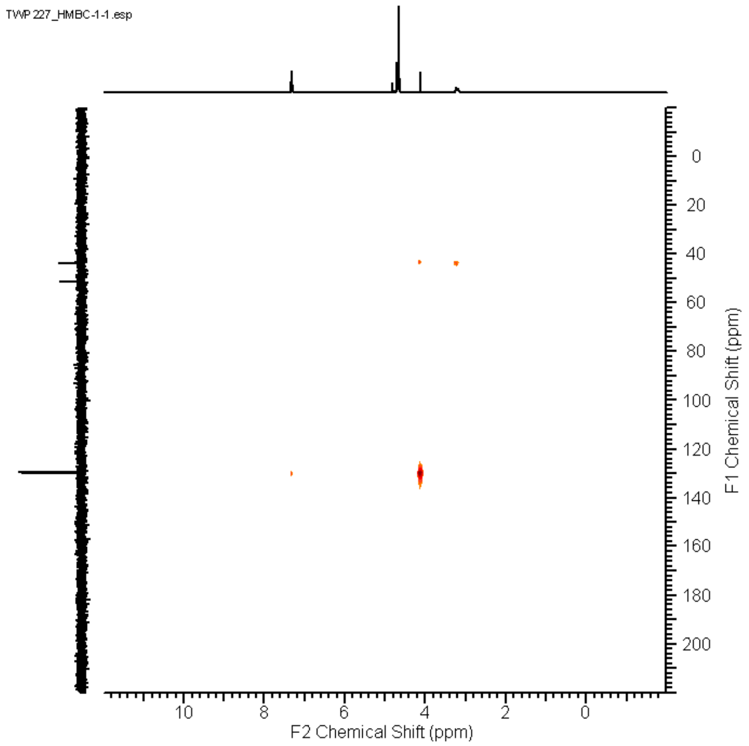

Figure S5: HMBC of **2** (D_2O, 298 K)

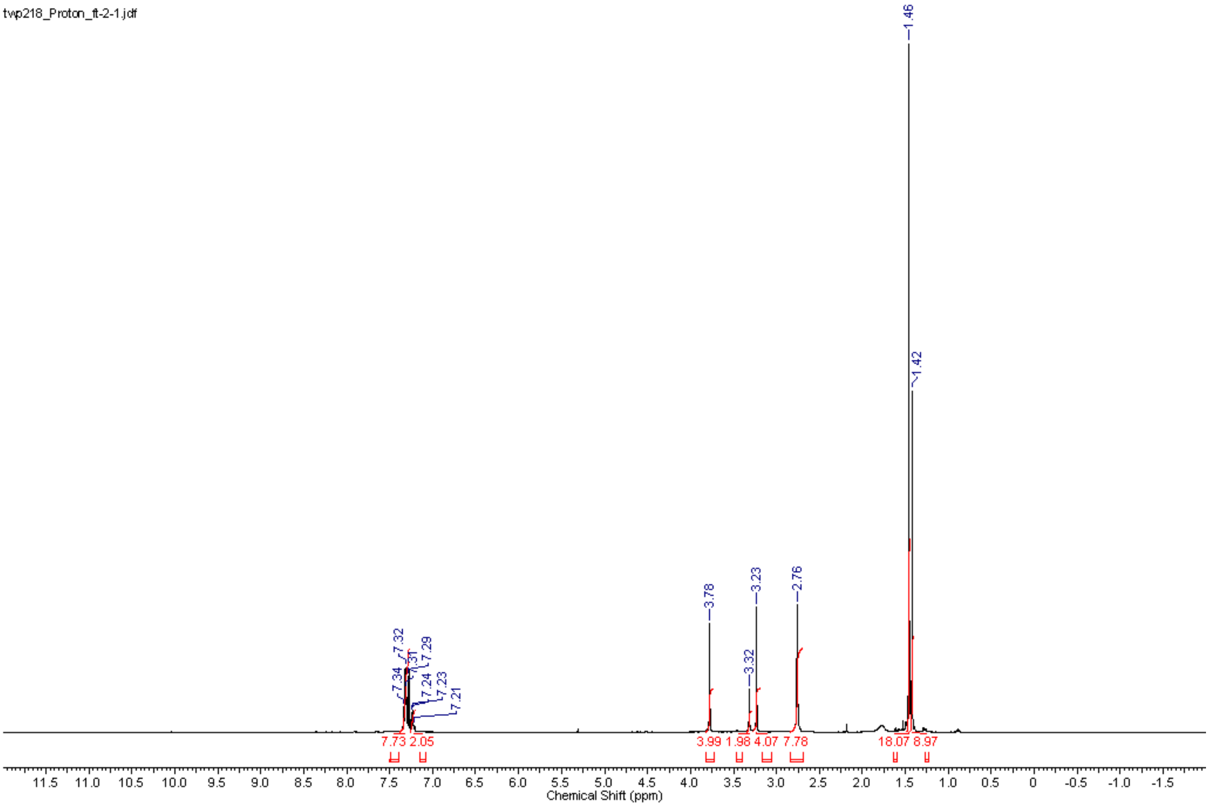

Figure S6: ^{1}H NMR of **3** (400 MHz, $CDCl_3$, 298 K)

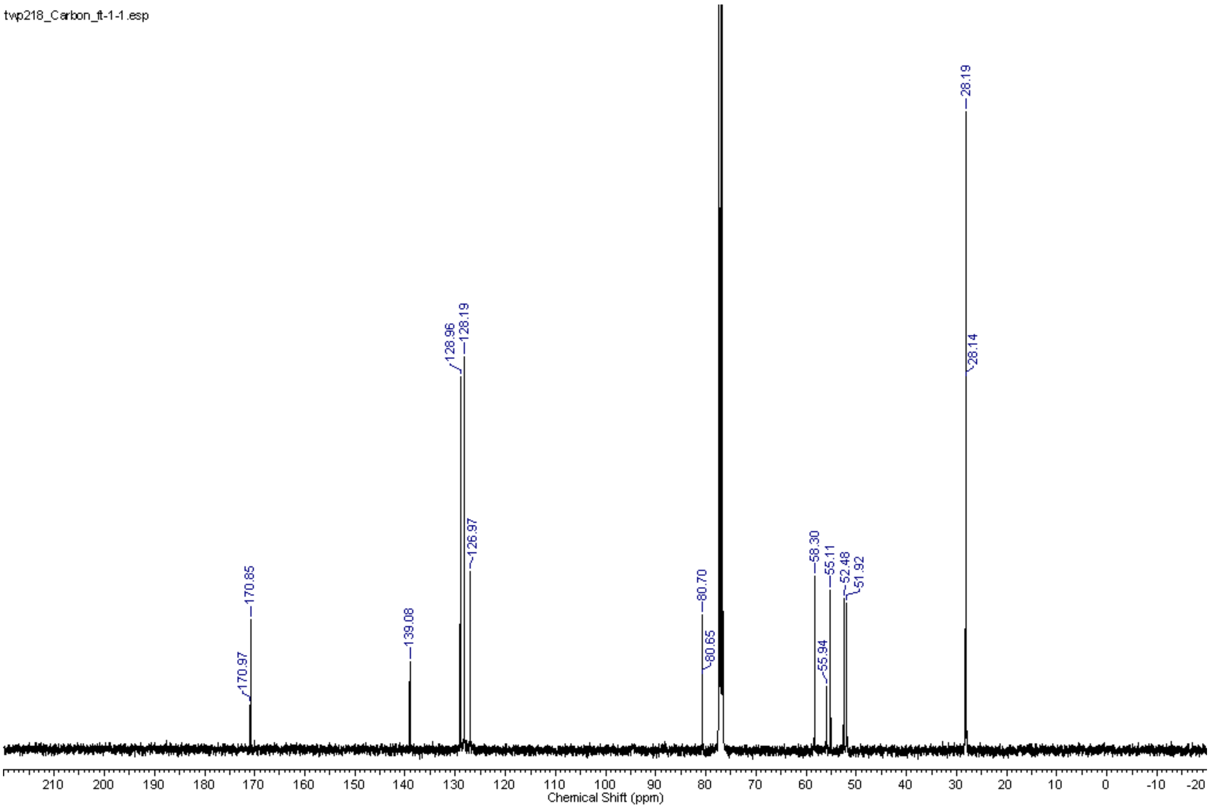

Figure S7: $^{13}C\{^{1}H\}$ NMR of **3** (100 MHz, $CDCl_3$, 298 K)

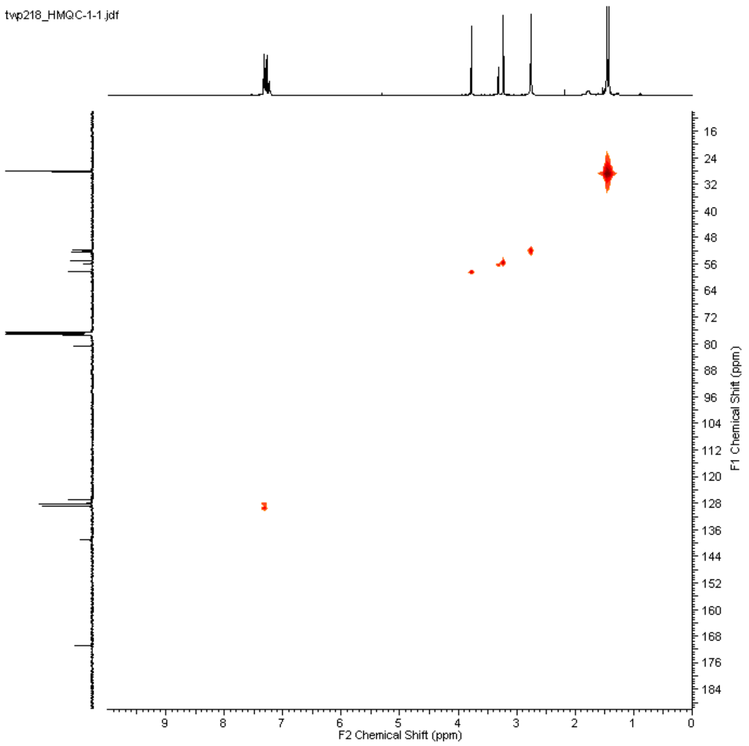

Figure S8: HMQC of **3** ($CDCl_3$, 298 K)

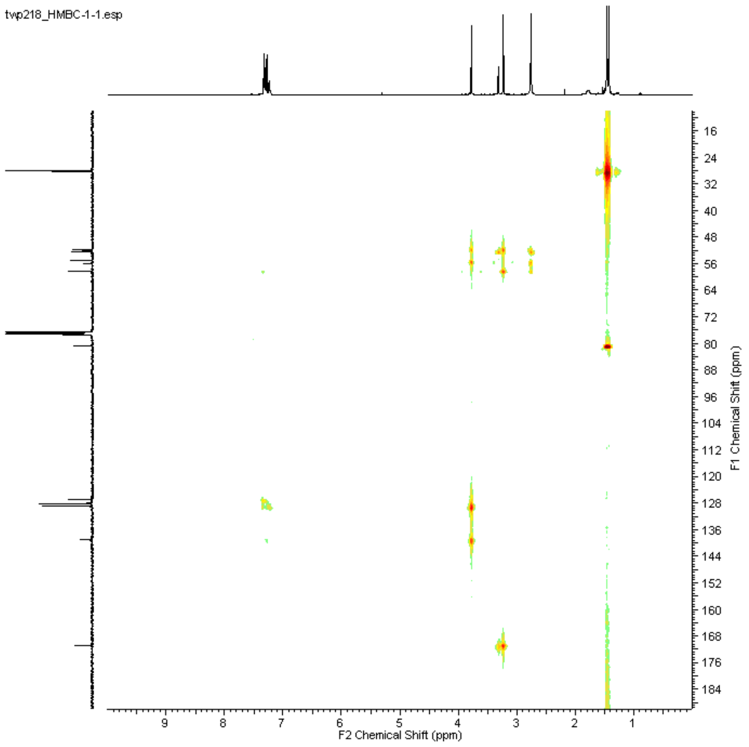

Figure S9: HMBC of **3** ($CDCl_3$, 298 K)

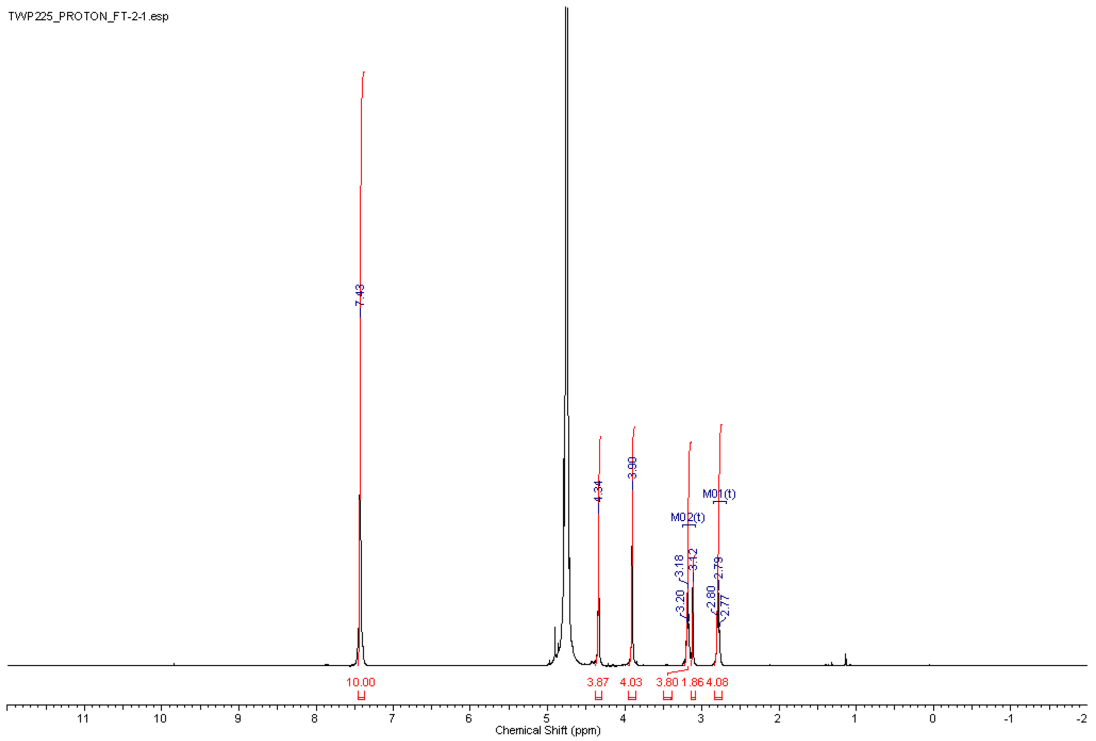

Figure S10: ^{1}H NMR of **Bn$_2$DT3A** (400 MHz, D_2O, 298 K)

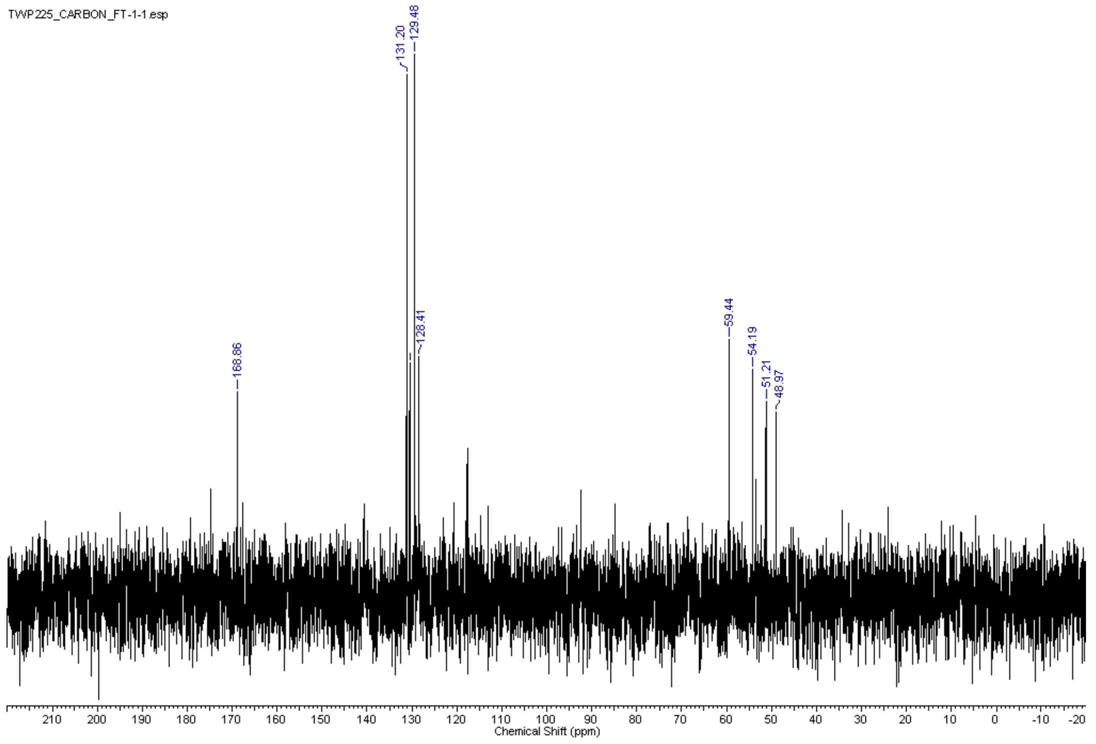

Figure S11: ^{13}C{^{1}H} NMR of **Bn$_2$DT3A** (100 MHz, D_2O, 298 K)

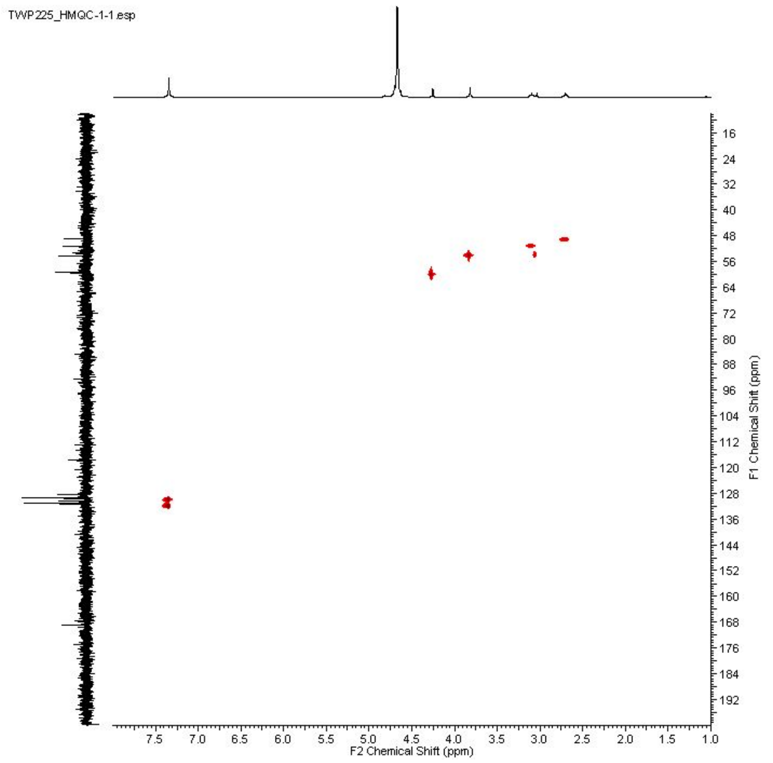

Figure S12: HMQC of **Bn$_2$DT3A** (D_2O, 298 K)

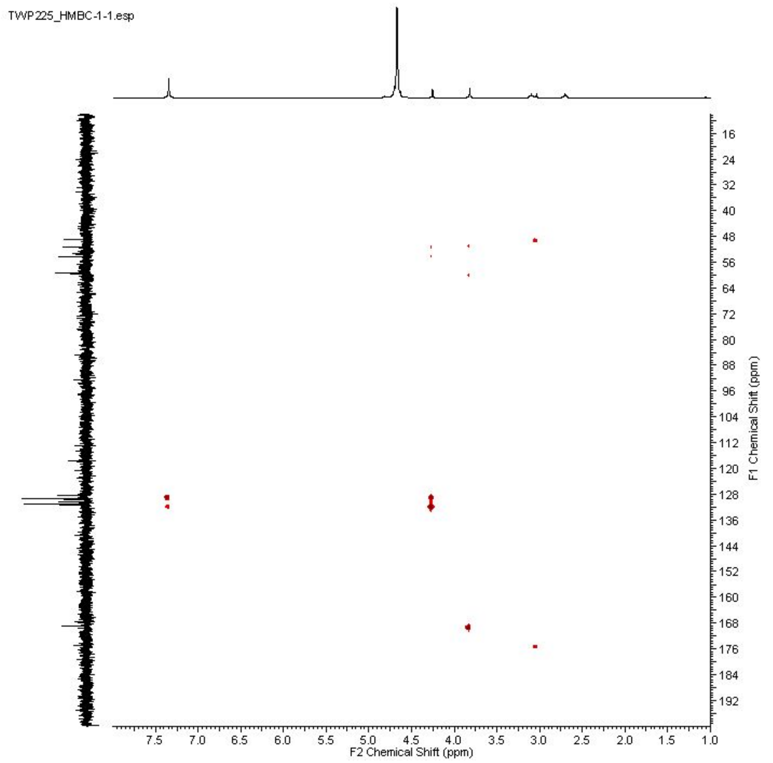

Figure S13: HMBC of **Bn$_2$DT3A** (D_2O, 298 K)

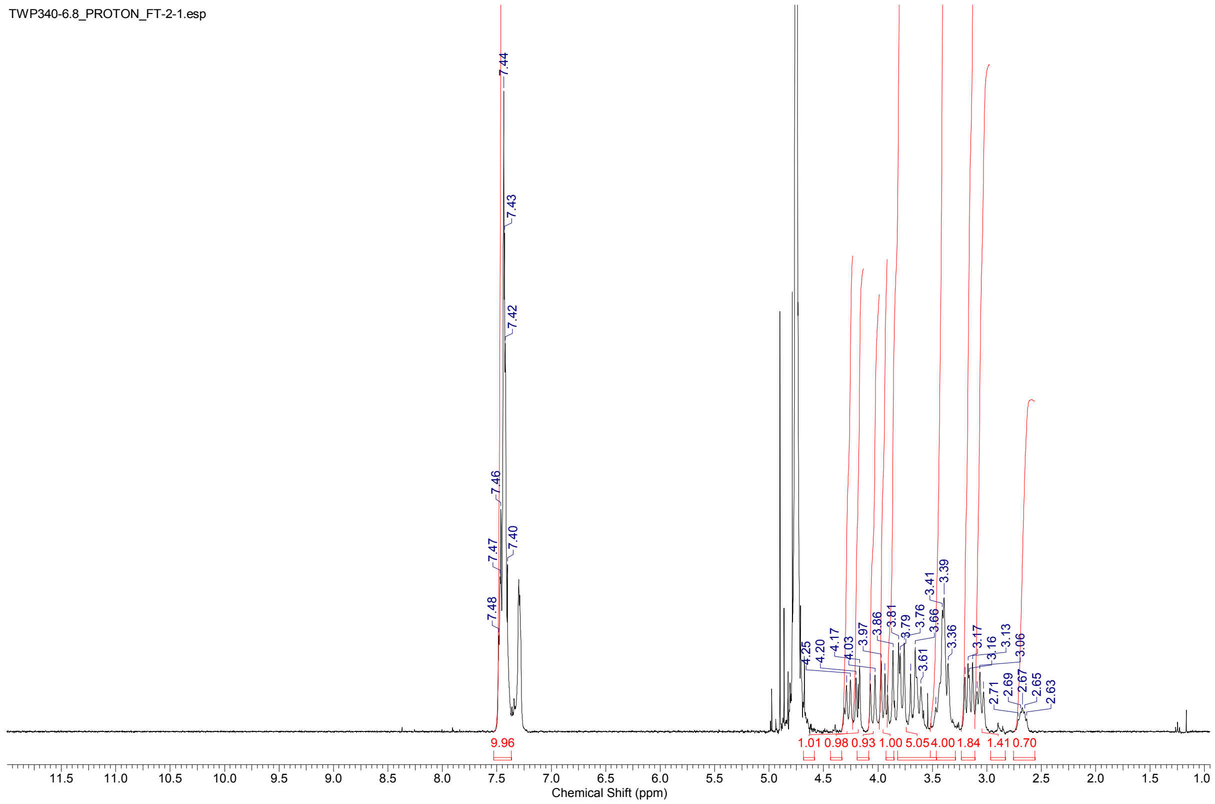

Figure S14: ^{1}H NMR of [Ga(**Bn$_2$DT3A**)(OH)]$^{-}$ (400 MHz, D_2O, pD = 6.8, 298 K)

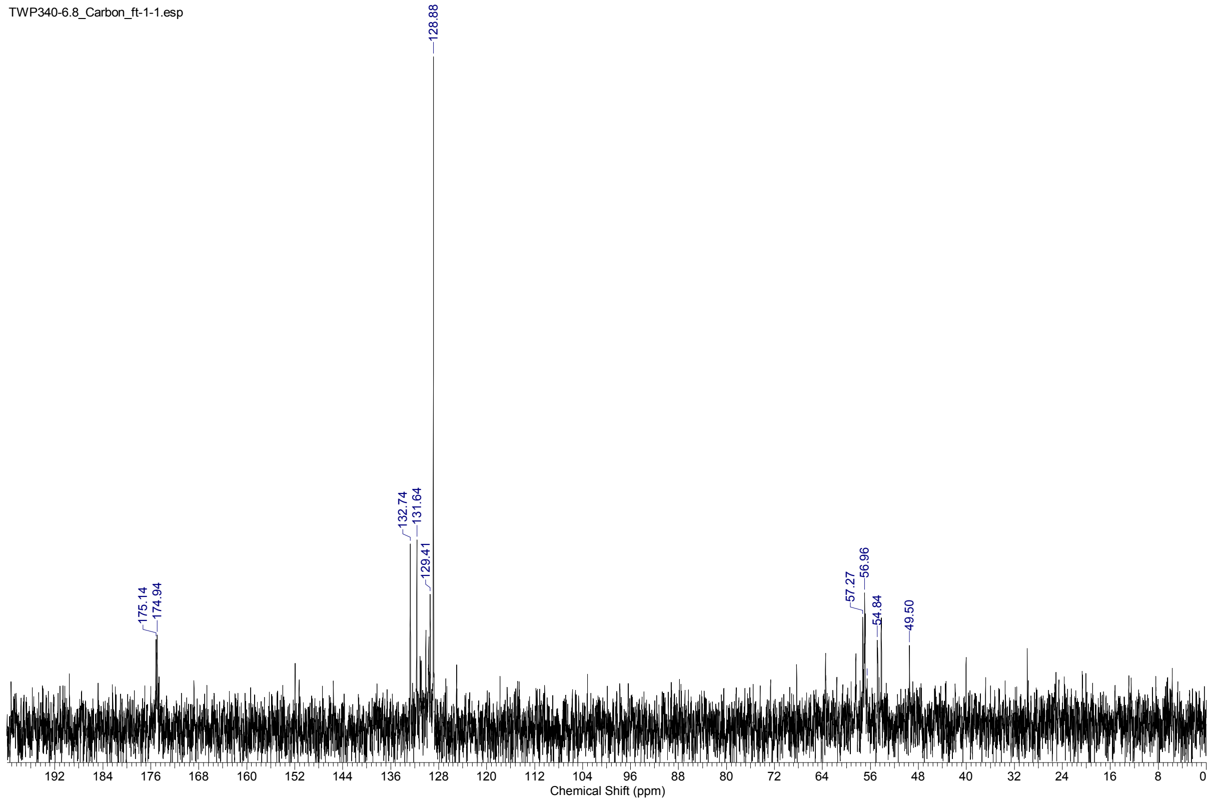

Figure S15: $^{13}C\{^{1}H\}$ NMR of [Ga(**Bn$_2$DT3A**)(OH)]$^{-}$ (100 MHz, D_2O, pD = 6.8, 298 K)

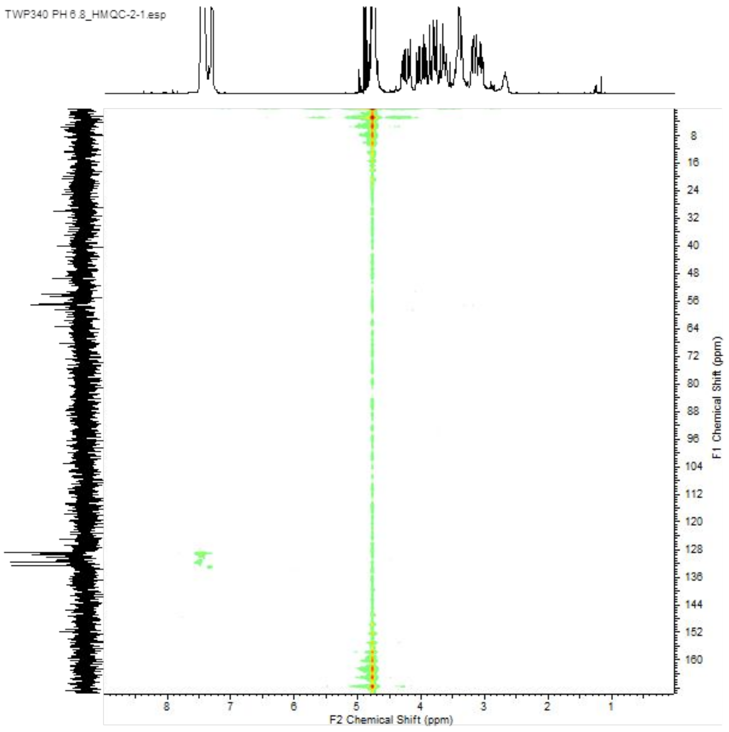

Figure S16: HMQC of [Ga(**Bn$_2$DT3A**)(OH)]$^{-}$ (D_2O, pD = 6.8, 298 K)

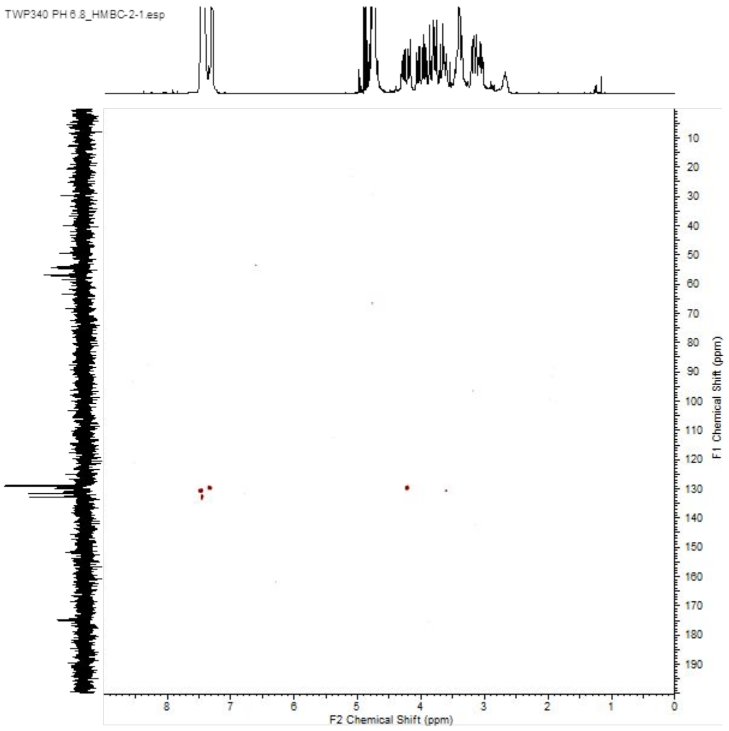

Figure S17: HMBC of [Ga(**Bn$_2$DT3A**)(OH)]$^-$ (D_2O, pD = 6.8, 298 K)

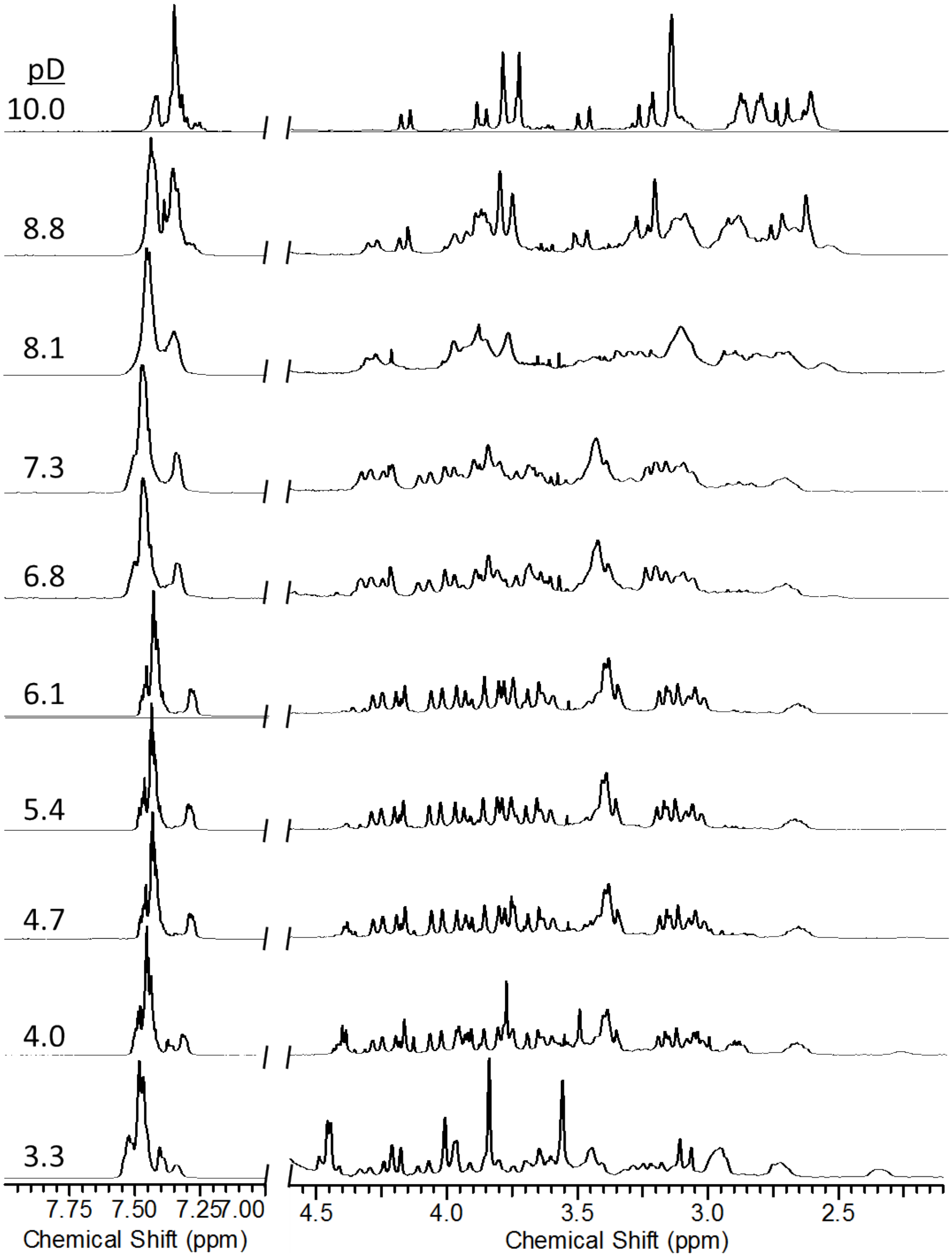

Figure S18: ^{1}H NMR spectra of [Ga(**Bn_2DT3A**)] recorded at indicated pD values (400 MHz, D_2O, 298 K)

3. Radiolabelling Data

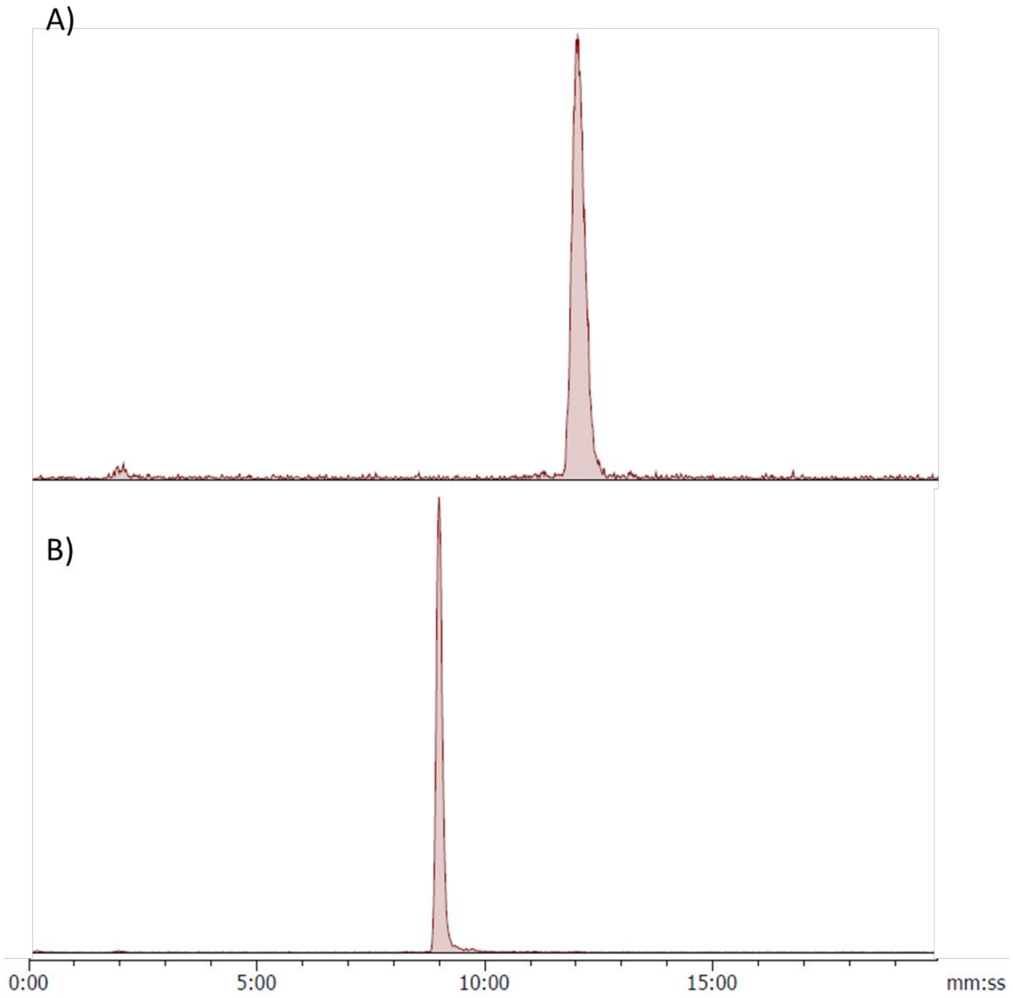

Figure S19: Radio-HPLC chromatograms of reinjected fractions isolated products A) [^{68}Ga][Ga(**Bn$_2$DT3A**)], B) [^{68}Ga][Ga(**Bn$_2$DT3A**)(OH)]$^{-}$

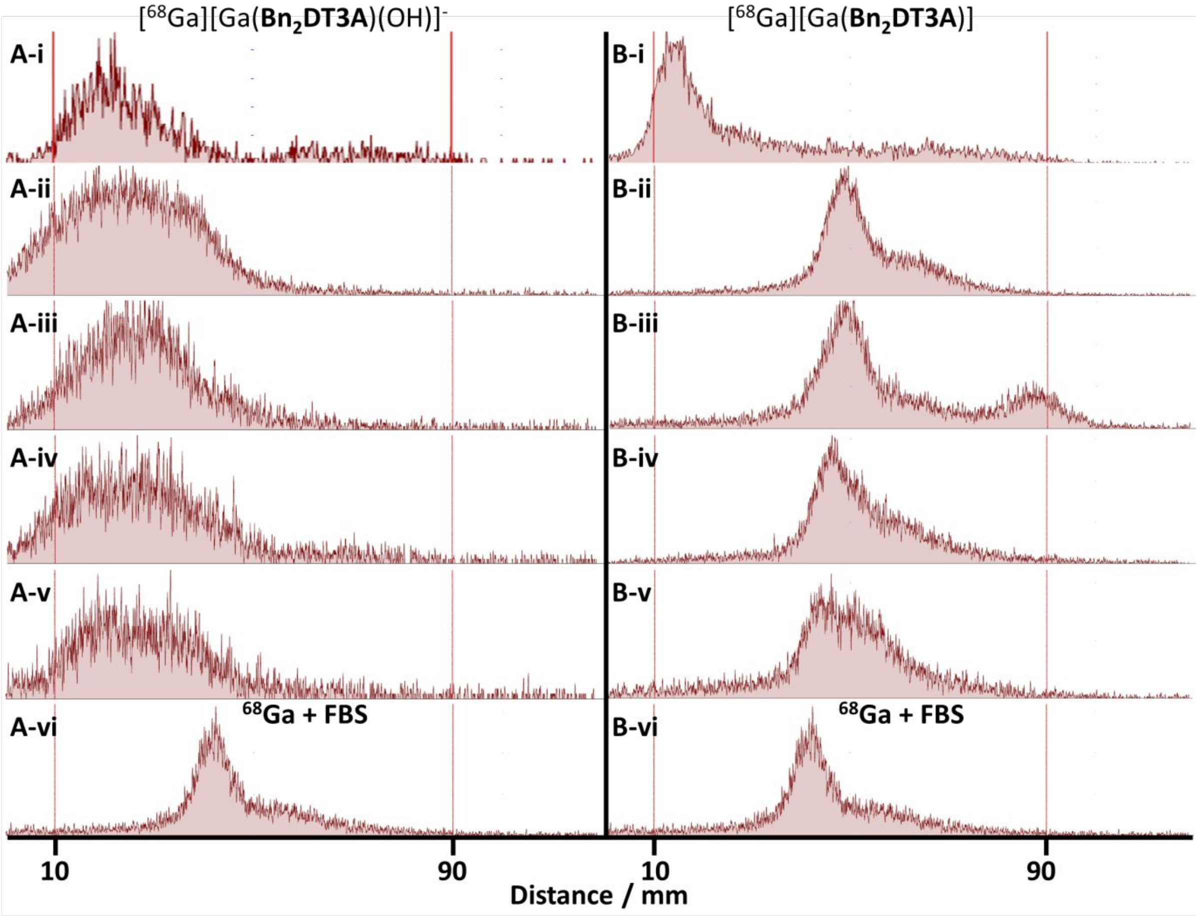

Figure S20: Stability of species formed in radiolabelling of **Bn$_2$DT3A** with ^{68}Ga following isolation. A) [^{68}Ga][Ga(**Bn$_2$DT3A**)(OH)]$^-$ B) [^{68}Ga][Ga(**Bn$_2$DT3A**)]. i) isolated species. ii) After 30 min incubation with FBS at 37 ^{o}C. iii) after 60 min incubation. iv) 90 min incubation. v) 120 min incubation. vi) [^{68}Ga][GaCl$_3$] incubated with FBS at 37 ^{o}C.

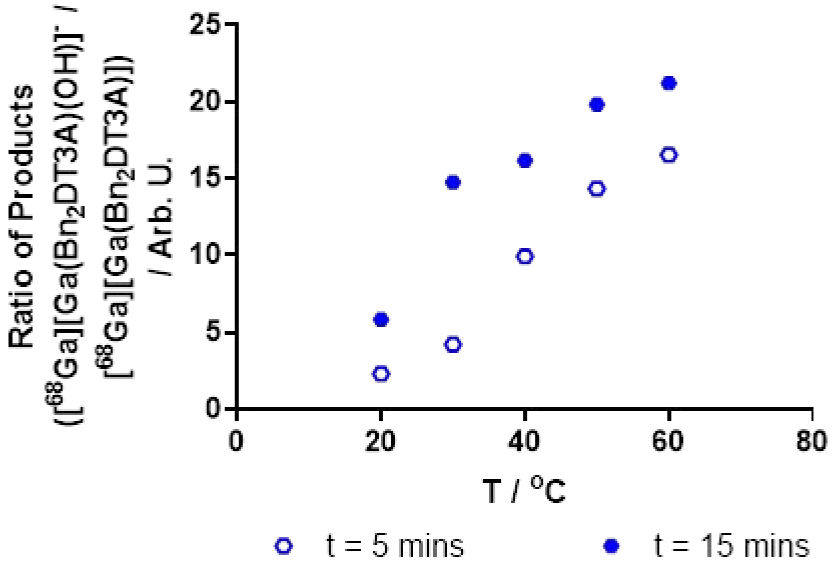

Figure S21: Effect of temperature on the ratio of products formed during radiolabelling of

Bn_2DT3A with ^{68}Ga. [**Bn_2DT3A**] = 100 µM, pH = 7.4, I = PBS

4. Potentiometry Data

Table S1: Overall protonation constants of ligands and stability constants (logβ_{HLM} of complexes. (T = 25 °C, I = 0.1 M NMe_4Cl) as determined by treatment of the equilibrium data. Charges are omitted. The stability constants corresponding to the formation of [Cu(HL)] were determined without ionic strength control.

Equilibrium	**Bn_2DT3A**
$H^+ + L \leftrightarrow HL$	9.70(1)
$2\ H^+ + L \leftrightarrow H_2L$	17.18(1)
$3\ H^+ + L \leftrightarrow H_3L$	20.52(1)
$4\ H^+ + L \leftrightarrow H_4L$	22.02(2)
$5\ H^+ + L \leftrightarrow H_5L$	23.42(2)
$Ga + L \leftrightarrow [Ga(L)]$	18.25(3)
$Ga + H^+ + L \leftrightarrow [Ga(HL)]$	20.98(3)
$Ga + H_2O + L \leftrightarrow [Ga(L)(OH)] + H^+$	12.93(2)
$Ga + 2\ H_2O + L \leftrightarrow [Ga(L)(OH)_2] + 2\ H^+$	4.72(2)
$Cu + L \leftrightarrow [Cu(L)]$	18.9(1)
$Cu + H^+ + L \leftrightarrow [Cu(HL)]$	21.7(1)
$Zn + L \leftrightarrow [Zn(L)]$	14.12(3)
$Zn + H^+ + L \leftrightarrow [Zn(HL)]$	18.28(1)

$Zn + H_2O + L \leftrightarrow [Zn(L)(OH)] + H^+$	2.06(4)

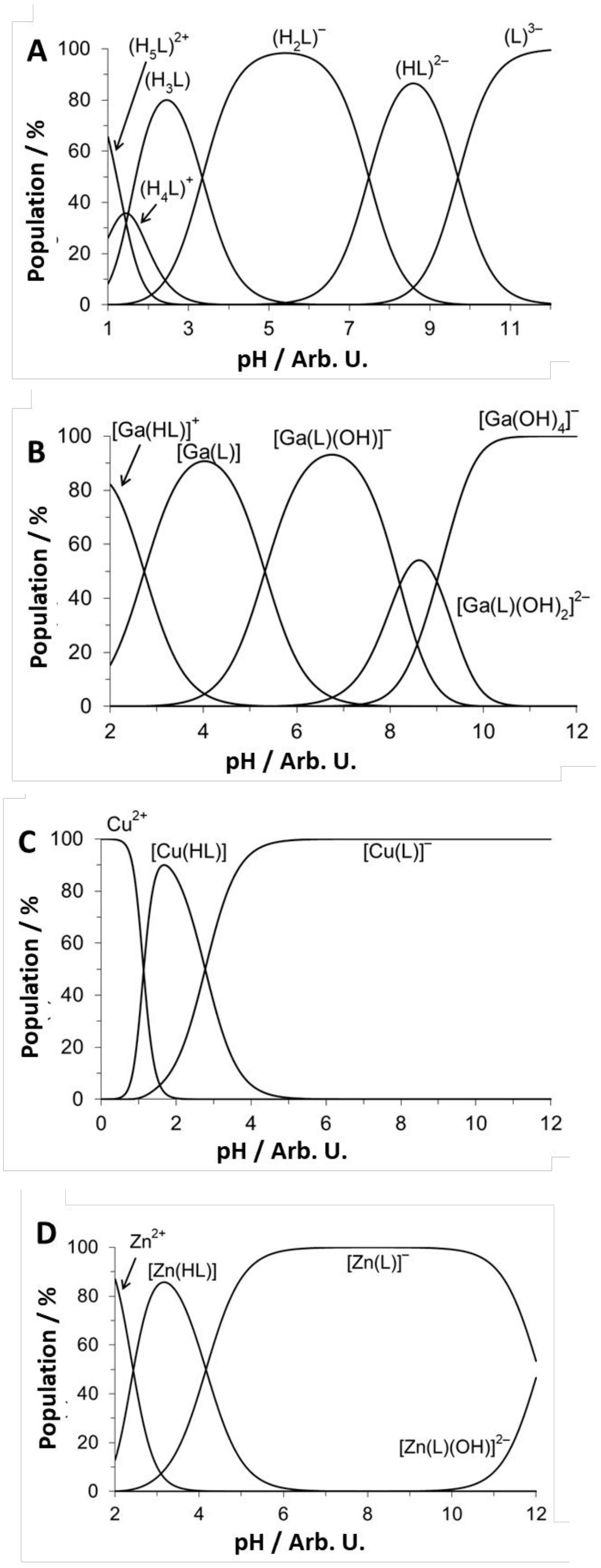

Figure S22: Distribution diagrams for A) **Bn_2DT3A** and B) Ga(III)-**Bn_2DT3A**, C) Cu(II)-**Bn_2DT3A** and D) Zn(II)-**Bn_2DT3A** systems. (T = 25 °C, I = 0.1 M NMe_4Cl, [L] = 4 μM, [M]

= 2 μM).

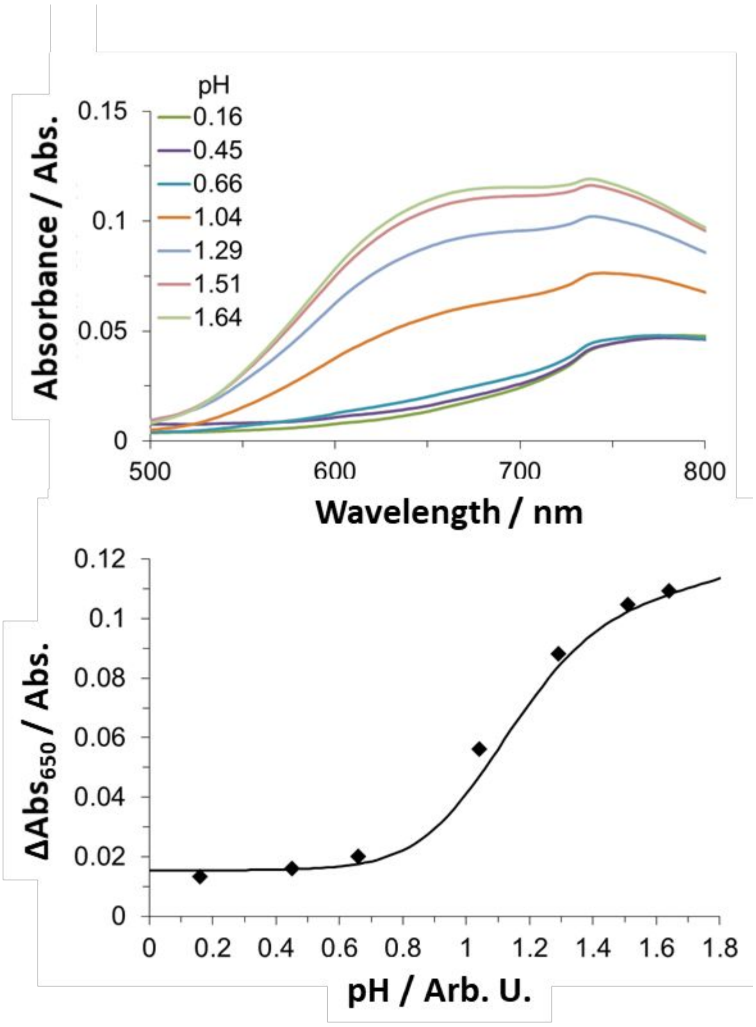

Figure S23: UV-Vis titration of Cu(II)-**Bn$_2$DT3A** system. Top: UV-Vis spectra variation with pH. Bottom: Difference of absorbance at 650 nm, ΔAbs, used to evaluate results. The line corresponds to the best fit. (*T* = 25 °C, [L] = [M] = 0.01 mM)

5. Crystallography Data

The compound crystallised as well-formed colourless blocks. An appropriate crystal of size 0.2 × 0.15 × 0.1 mm^3 was selected from the reaction mixture and examined using at 100 K using a STOE STADI VARI 200K Pilatus diffractometer operating with Cu-radiation (Xenocs, Cu-Genix 3D HF Microfocus source). The crystal was mounted using a MiTeGen MicroMount with perfluorinated ether. A set of diffraction data were collected using the rotation method (ω-scans with 0.5° frames). Data were scaled and treated for the effects of absorption using standard techniques with STOE LANA.

The structure was solved using dual-space methods within SHELXT and structure refinement based on F^2 was carried out with SHELXL-2018/1. Non-hydrogen atoms of the metal complex were refined using anisotropic displacement parameters. Hydrogen atoms were placed using a riding model. The structure is non-centric; the Flack parameter was refined using 1665 quotients and found to be zero within error. Final R-factors indicate a good fit to the observed data: (all data) R1 = 0.0771, $wR(F^2)$ = 0.1870.

There is evidence for a small amount of poorly-resolved water within the structure. The structure has formula [Ga(**Bn$_2$DT3A**)]·0.33H_2O. An ORTEP diagram for the asymmetric unit is shown below and tables detailing the refinement and crystal structure follow.

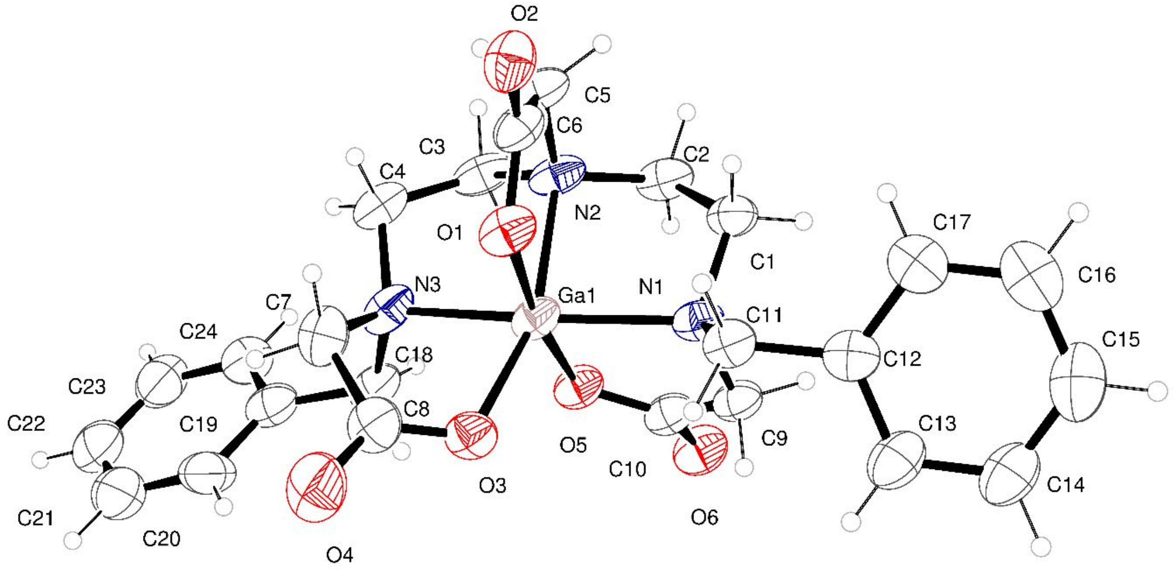

Figure S24: ORTEP representation of molecular structure of [Ga(**Bn$_2$DT3A**)] obtained by single crystal X-ray crystallography (drawn as 30% probability ellipsoids) of a crystal grown at an acidic pH. CCDC Reference: 1864389.

Table S2: Crystal data and structure refinement for [Ga(**Bn_2DT3A**)] for crystal grown at acidic pH. CCDC Reference: 1864389

Parameter	[Ga(**Bn_2DT3A**)]	
Empirical formula	C_{24} $H_{28.66}$ Ga N_3 $O_{6.33}$	
Structural formula	[Ga(**Bn_2DT3A**)]·0.33H_2O	
Formula weight	529.55	
Temperature	100(2) K	
Wavelength	1.54186 Å	
Crystal system	Trigonal	
Space group	R3 (hexagonal setting)	
Unit cell dimensions	*a* = 24.5666(5) Å	α = 90°.
	b = 24.5666(5) Å	β = 90°.
	c = 10.0351(2) Å	γ = 120°.
Volume	5245.0(2) $Å^3$	
Z	9	
Density (calculated)	1.509 Mg/m^3	

Absorption coefficient	2.027 mm^{-1}
$F(000)$	2472
Crystal size	0.200 × 0.150 × 0.100 mm^3
Theta range for data collection	4.873 to 69.572°.
Index ranges	$-29 \leq h \leq 21$, $-22 \leq k \leq 29$, $-9 \leq l \leq 12$
Reflections collected	15590
Independent reflections	3910 [R(int) = 0.0264]
Completeness to theta = 67.686°	99.70%
Absorption correction	Semi-empirical from equivalents
Max. and min. transmission	0.6268 and 0.0495
Refinement method	Full-matrix least-squares on F^2
Data / restraints / parameters	3910 / 1 / 287
Goodness-of-fit on F^2	1.102
Final R indices [I>2sigma(I)]	$R1 = 0.0758$, $wR2 = 0.1849$
R indices (all data)	$R1 = 0.0771$, $wR2 = 0.1870$

Absolute structure parameter	0.00(4)
Extinction coefficient	none
Largest diff. peak and hole	1.830 and -0.447 e Å^{-3}

Crystals were grown at two further pHs (5.3 and 6.8). The crystal structure obtained from these two preparations was the same. The crystals were studied at the same temperature as the previously described [Ga(**Bn_2DT3A**)] crystal but the structures of these are different from it. Data were collected at the EPSRC National Crystallography Service, Southampton, UK, on a Rigaku XtaLAB diffractometer operating with a Mo rotating anode source. Routine procedures for data processing and structure solution/refinement were carried out. The structure of the crystal collected from the solution at pH 6.8 is shown below (Figure S25).

The same molecule is present, namely [Ga(**Bn_2DT3A**)] and this is also a hydrate, with formula [Ga(**Bn_2DT3A**)]·0.25H_2O. There are four unique complexes in the asymmetric unit in the centrosymmetric space group P-1. Three of these complexes have very similar conformation (Ga1, Ga2, Ga3, in figure S25 below) but the fourth (Ga4) is different. This is most noticeable in the torsion angle at the nitrogen bearing the benzyl group. It is highlighted in the overlay figures below. (Figure S26)

The structure of this second form was completed using a solvent mask implemented in Olex2. It was not possible to find a suitable model for the disordered water present. The amount of water present identified by the use of the solvent mask was completely consistent with that identified as two disorder components from the electron density map.

CCDC number 2125953 contains the structural data for this phase.

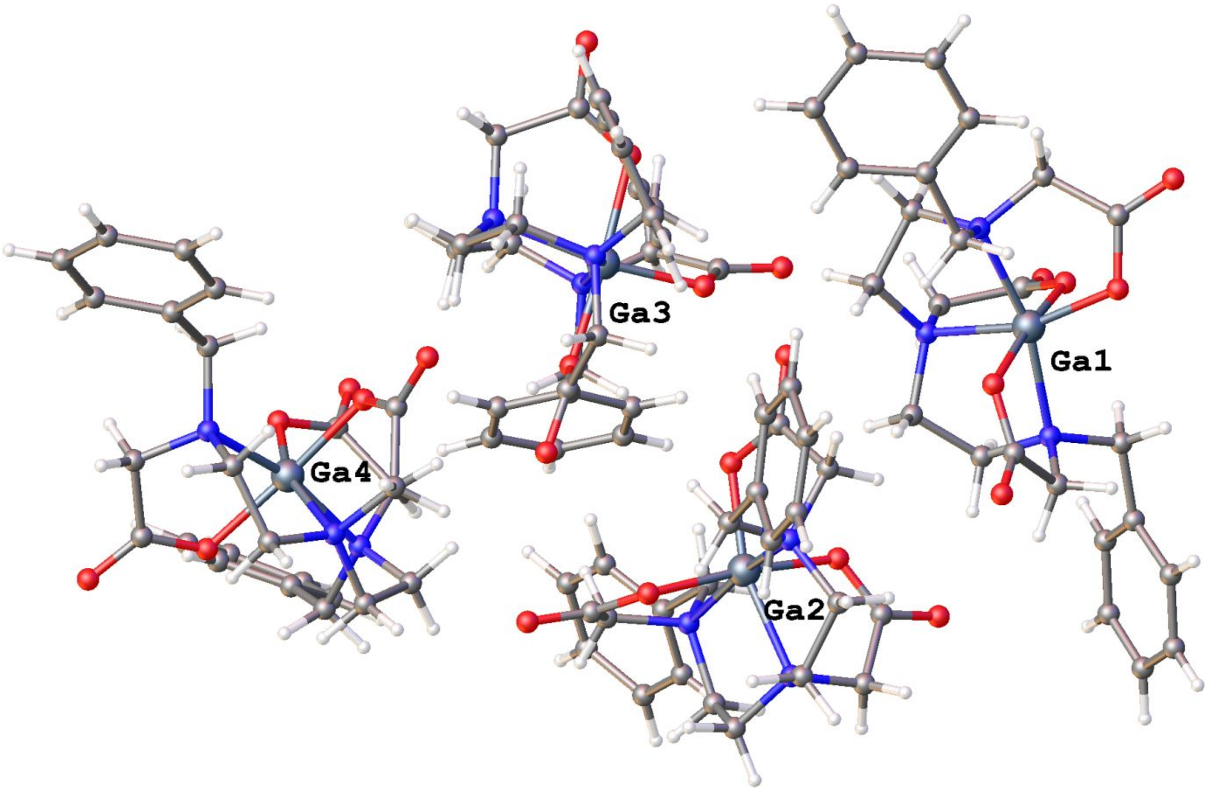

Figure S25: Molecular structure of [Ga(Bn_2DT3A)] from crystal grown at pH 6.8 determined by X-ray crystallography. Hydrogen atoms have been omitted for clarity. Colors: gallium (pale brown); carbon (gray); nitrogen (blue); oxygen (red).

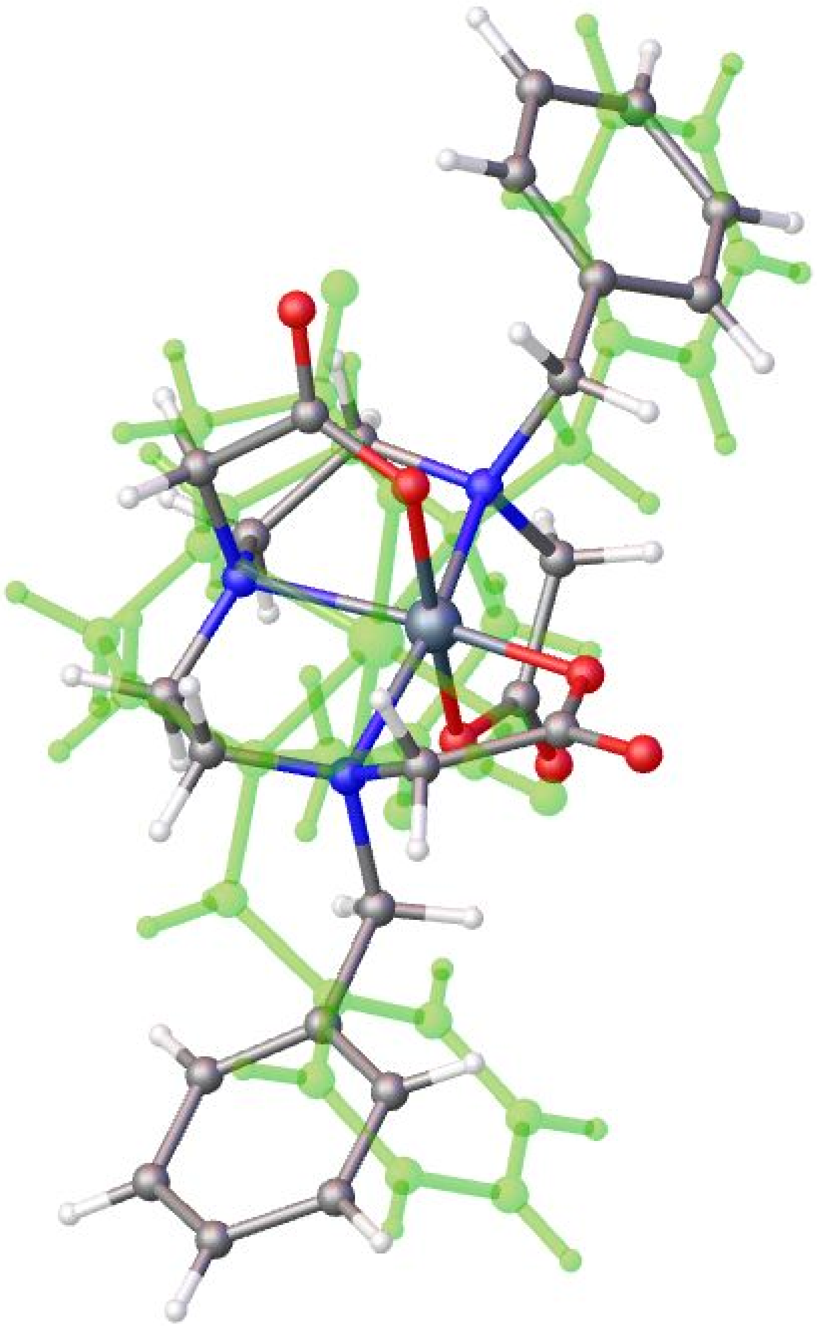

Figure S26: One view of complex of Ga4 overlayed on complex of Ga1. (Ga4 complex in green; Ga1 complex colored by atom)

Table S3: Crystal data and structure refinement for [Ga(**Bn_2DT3A**)] for crystal grown at pH 6.8. CCDC Reference: 2125953

Identification code	2018ncs0919s	
Empirical formula	$C_{24}GaH_{28.5}N_3O_{6.25}$	
Structural Formula	[Ga(**Bn_2DT3A**)] 0.25H_2O	
Formula weight	528.72	
Temperature/K	100	
Wavelength	0.71075 Å	
Crystal system	triclinic	
Space group	P-1	
Unit cell dimensions	a = 13.5522(3) Å	α = 98.200(2)°.
	b = 14.8380(4) Å	β = 104.254(2)°.
	c = 24.2353(7) Å	γ = 90.691(2)°.
Volume	4669.4(2) $Å^3$	
Z	8	

Density (calculated)	1.504 Mg/m^3
Absorption coefficient	1.225 mm^{-1}
F(000)	2196.0
Crystal size	0.12 × 0.1 × 0.01 mm^3
2Θ range for data collection	3.16 to 51.364°.
Index ranges	-16 ≤ h ≤ 15, -18 ≤ k ≤ 17, -29 ≤ l ≤ 29
Reflections collected	53772
Independent reflections	17580 [R_{int} = 0.1122, R_{sigma} = 0.1171]
Completeness to theta = 67.686°	99.1%
Absorption correction	Semi-empirical from equivalents
Max. and min. transmission	0.6268 and 0.0495
Refinement method	Full-matrix least-squares on F^2
Data / restraints / parameters	17580/1266/1225
Goodness-of-fit on F^2	1.032
Final R indices [I>2sigma(I)]	R_1 = 0.0887, wR_2 = 0.2048

R indices (all data)	R_1 = 0.1336, wR_2 = 0.2238
Extinction coefficient	none
Largest diff. peak and hole	1. 1.51 and -0.98 e $Å^{-3}$

6. In Vivo Data

a. [^{68}Ga][Ga(**Citrate**)] – 3 bed positions

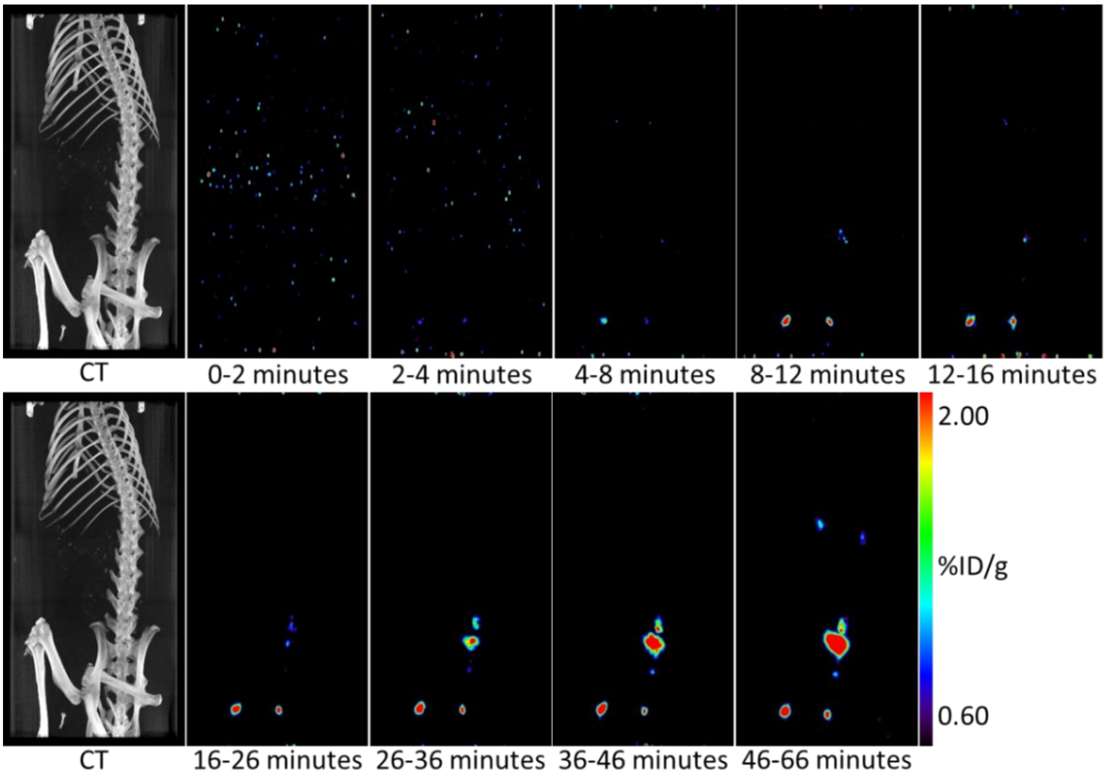

Figure S27: Coronal PET and CT scans of male rat injected with [^{68}Ga][Ga(**Citrate**)]$^{-}$.

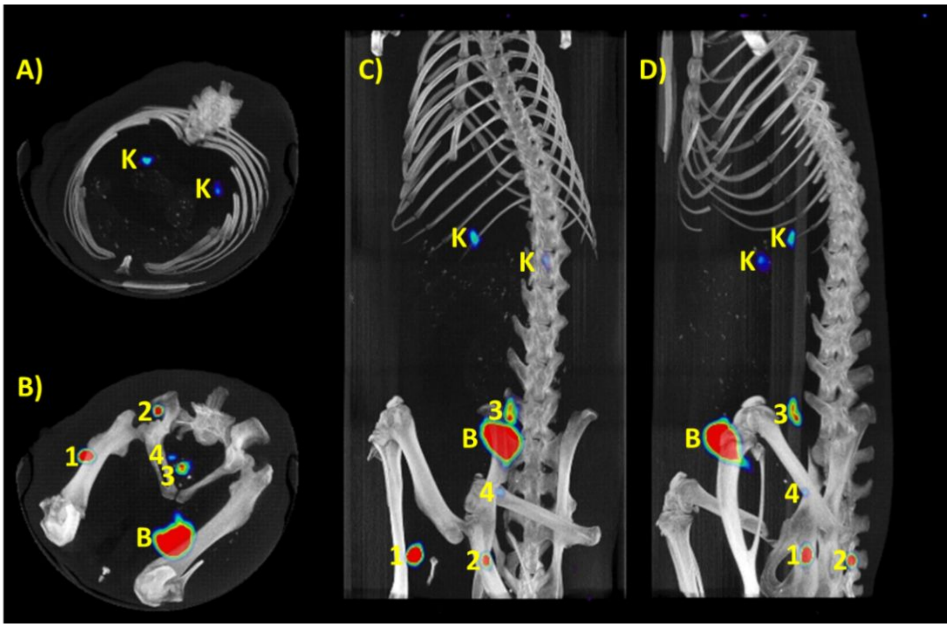

Figure S28: PET-CT fused scans of a rat 46–66 min after injection with [^{68}Ga][Ga(**Citrate**)]$^{-}$. A) Transverse projection of upper abdomen. B) Transverse projection of lower abdomen. C) Coronal projection. D) Sagittal projection. Areas of increased uptake are annotated. K = kidneys, B = bladder.

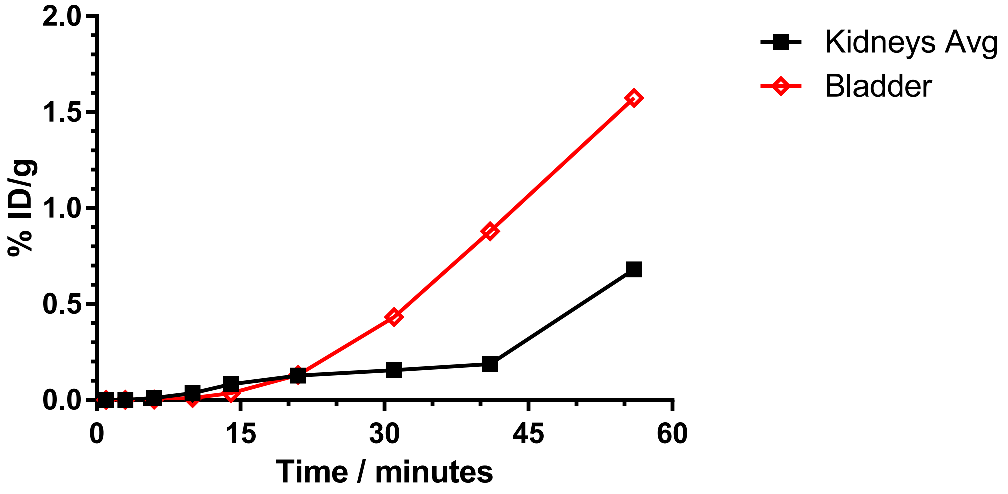

Figure S29: Activity time curves for selected organs following administration of [^{68}Ga][Ga(**Citrate**)]$^{-}$.

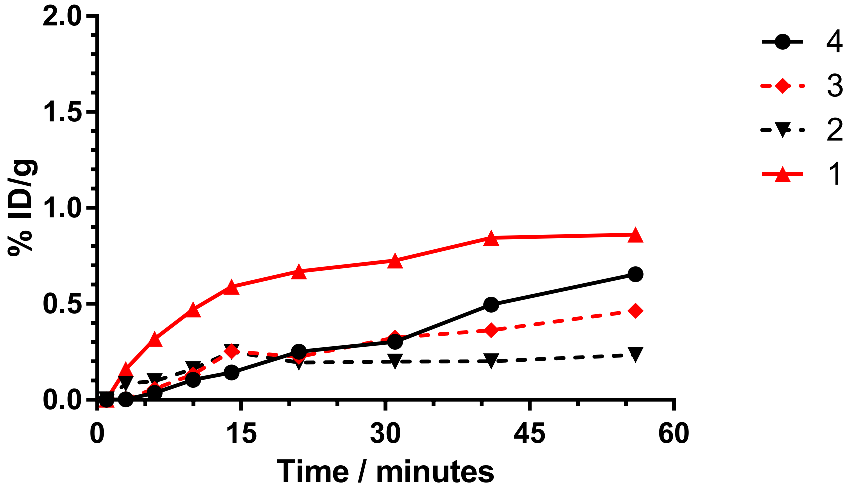

Figure S30: Activity-time curves for additional regions of increased uptake following administration of [^{68}Ga][Ga(**Citrate**)]$^{-}$.

b. [^{68}Ga][Ga(**Citrate**)] – 3 bed positions

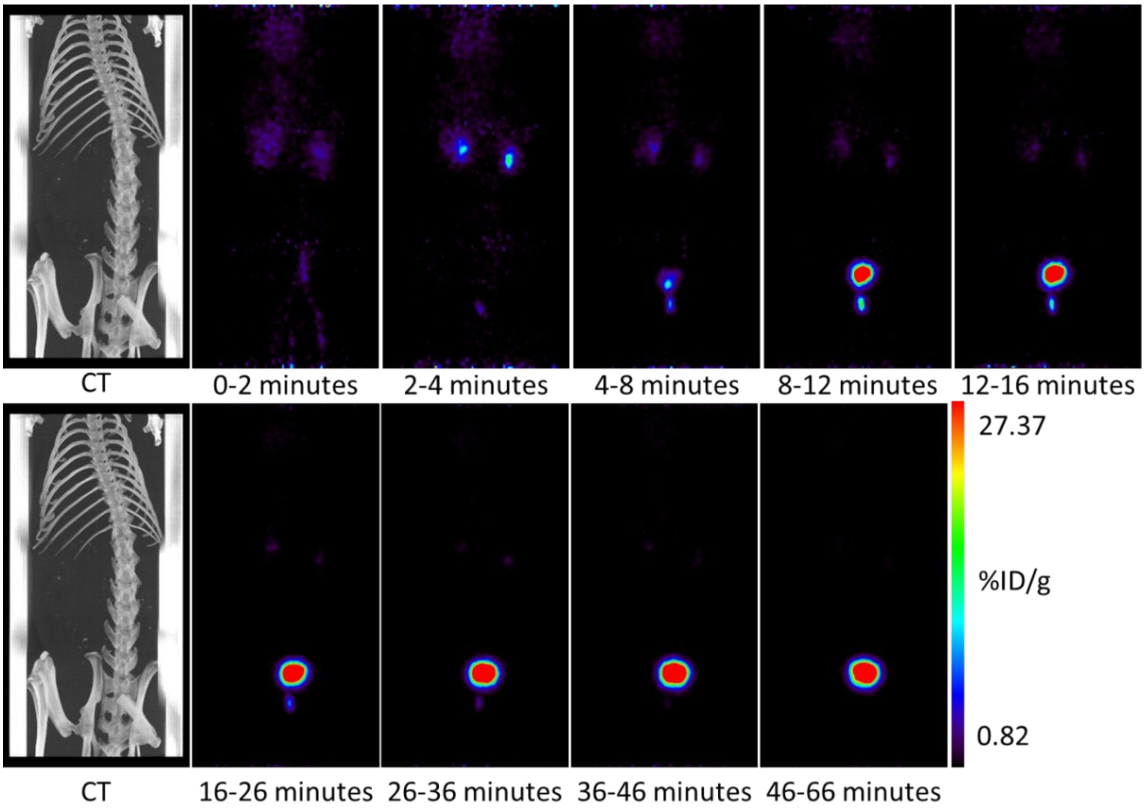

Figure S31: Coronal PET and CT scans of male rat injected with [^{68}Ga][Ga(**Citrate**)]$^{-}$.

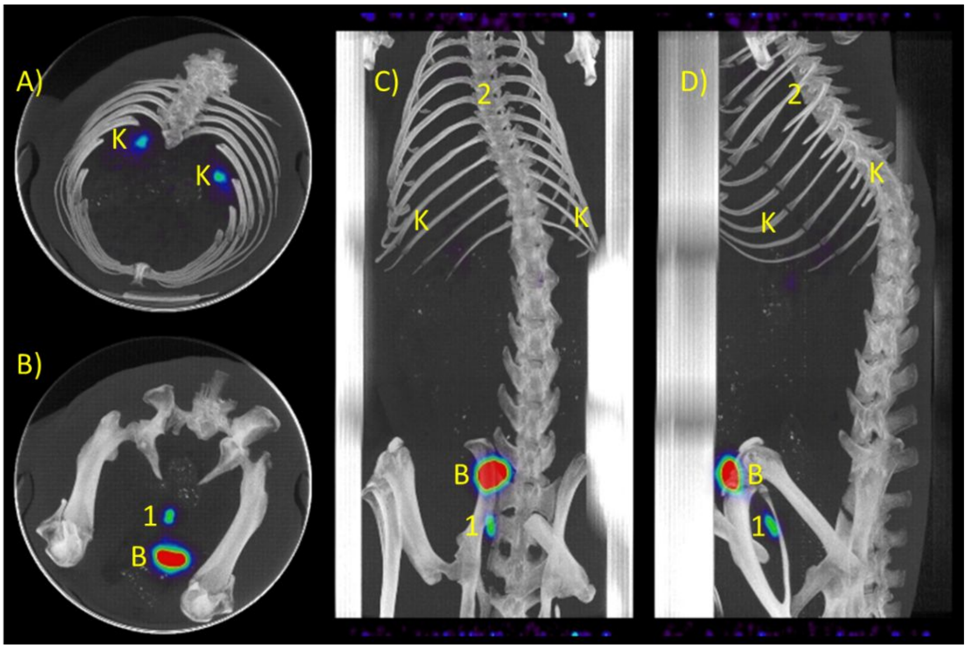

Figure S32: Fused PET-CT scans of a rat after injection with [^{68}Ga][Ga(**Citrate**)]$^{-}$. A) Transverse projection of upper abdomen 2–4 min after injection. B) Transverse projection of lower abdomen 12–16 min post injection. C) Coronal projection 12–16 min post injection. D) Sagittal projection 12–16 min post injection. Areas of increased uptake are annotated. K = kidneys, B = bladder.

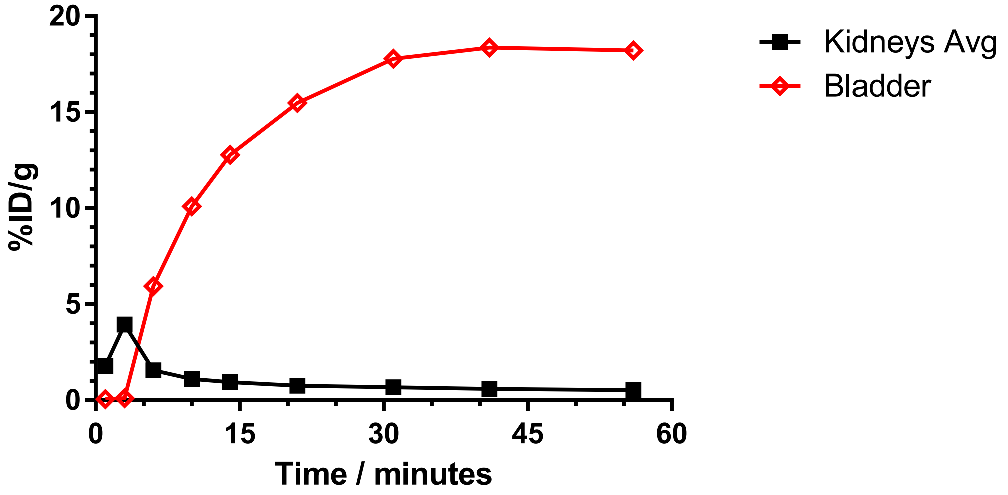

Figure S33: Activity time curves for selected organs following administration of

[^{68}Ga][Ga(**Citrate**)]$^{-}$.

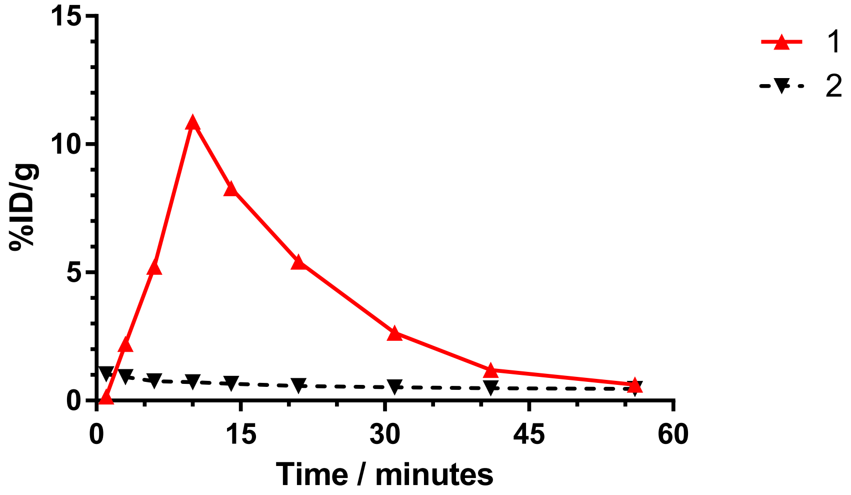

Figure S34: Activity-time curves for additional regions of increased uptake following

administration of [^{68}Ga][Ga(**Citrate**)]$^{-}$.

c. [^{68}Ga][Ga(**Bn$_2$DT3A**)(OH)]$^-$ – 2 bed positions

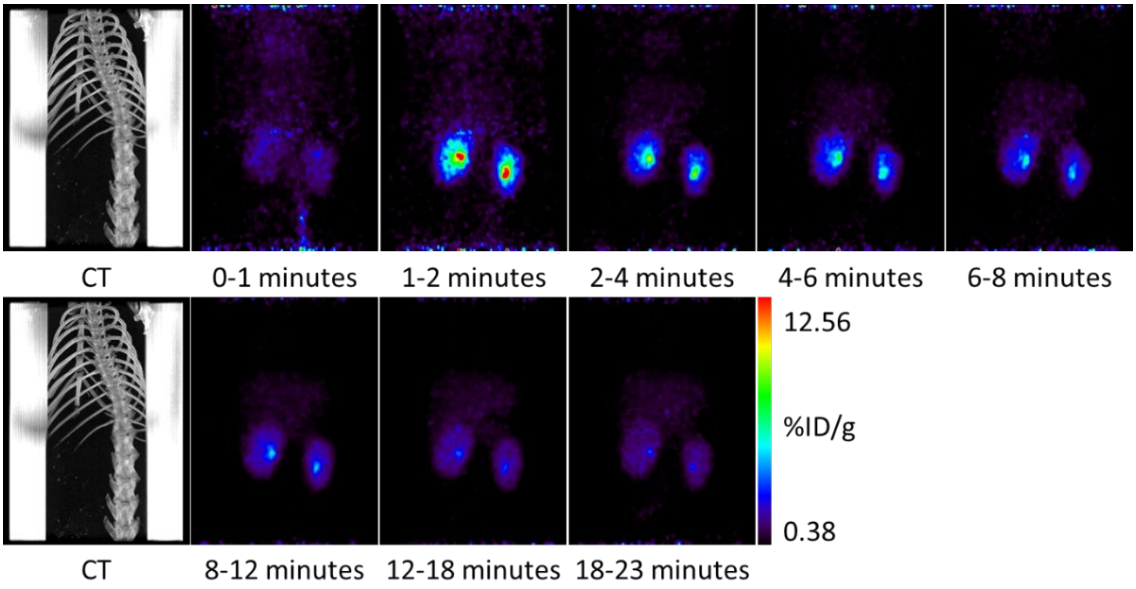

Figure S35: Coronal PET and CT scans of male rat injected with [^{68}Ga][Ga(**Bn$_2$DT3A**)(OH)]$^-$

.

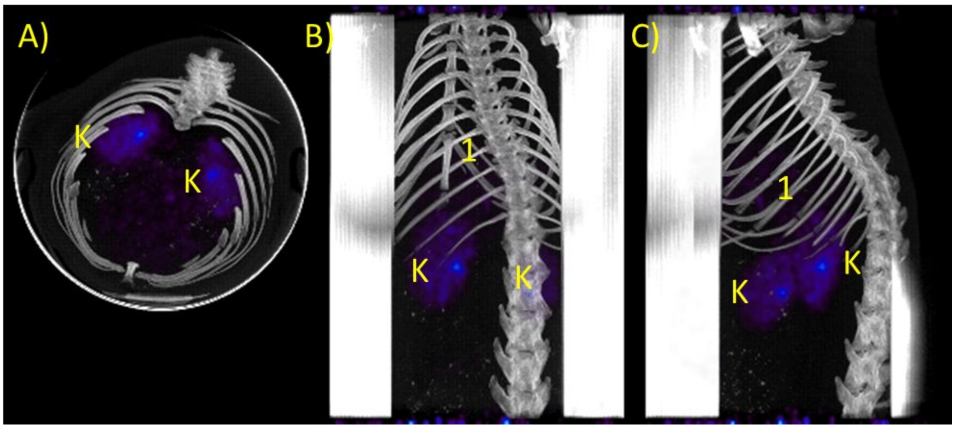

Figure S36: Fusion PET-CT scans of rat injected with [^{68}Ga][Ga(**Bn$_2$DT3A**)(OH)]$^{-}$ 18–23 min post injection. A) Transverse projection. B) Coronal projection. C) Sagittal projection. Areas of increased uptake are annotated. K = kidneys.

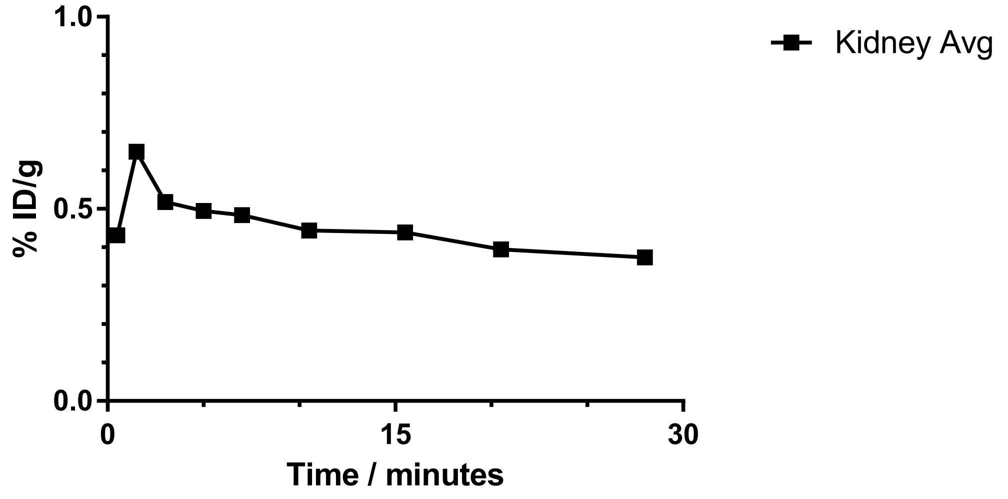

Figure S37: Activity time curves for selected organs following administration of [^{68}Ga][Ga(**Bn$_2$DT3A**)(OH)]$^{-}$.

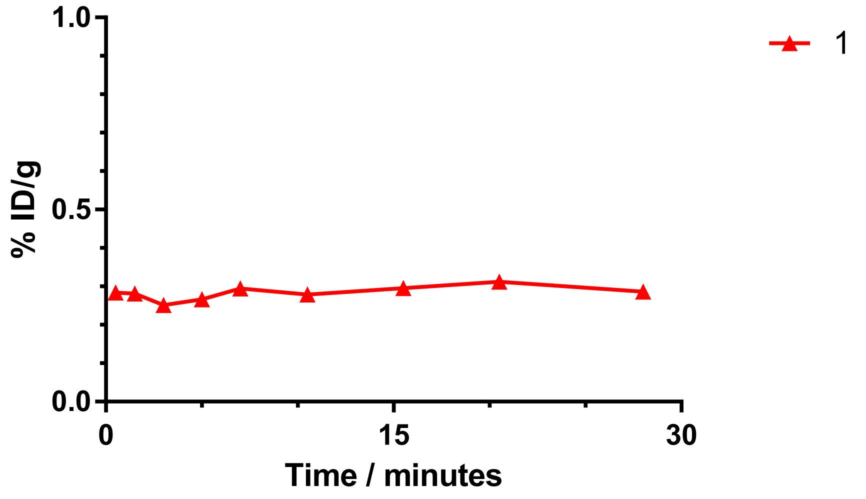

Figure S38: Activity-time curves for additional regions of increased uptake following administration of [^{68}Ga][Ga(**Bn$_2$DT3A**)(OH)]$^{-}$.

d. [^{68}Ga][Ga(**Bn$_2$DT3A**)(OH)]$^-$ – 3 bed positions

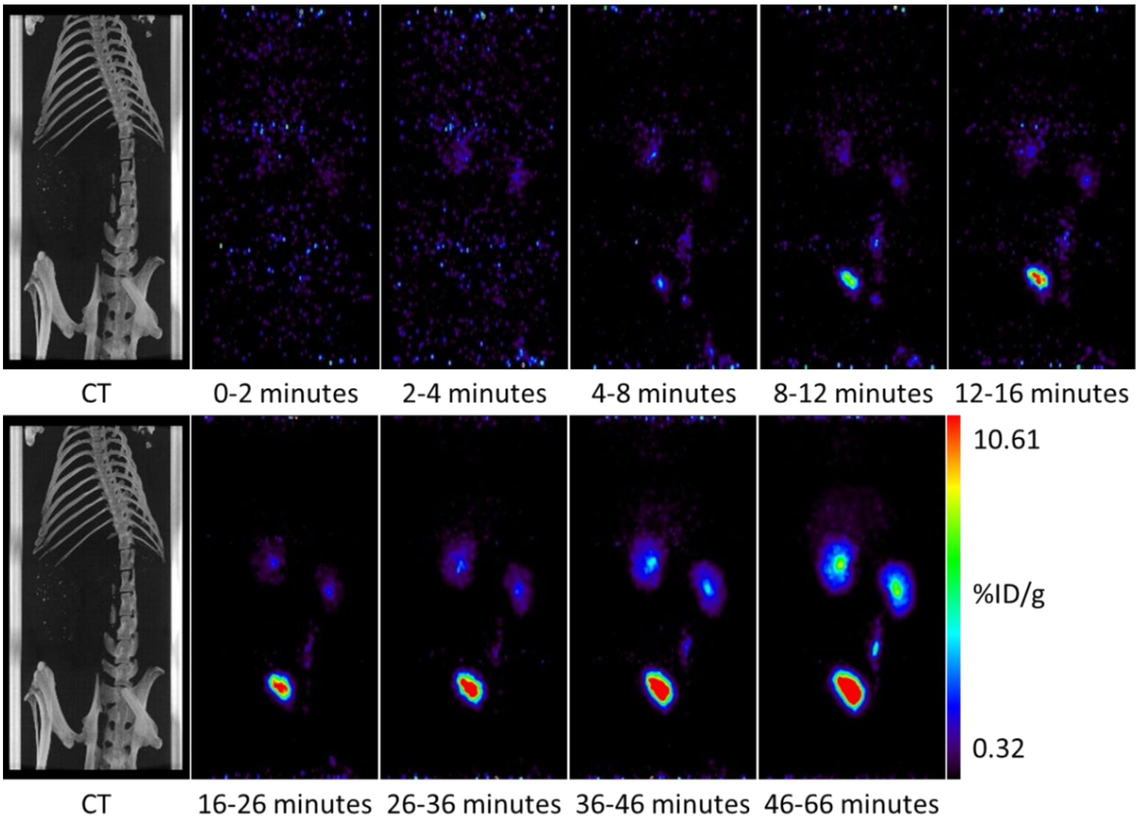

Figure S39: Coronal PET and CT scans of male rat injected with [^{68}Ga][Ga(**Bn$_2$DT3A**)(OH)]$^-$

.

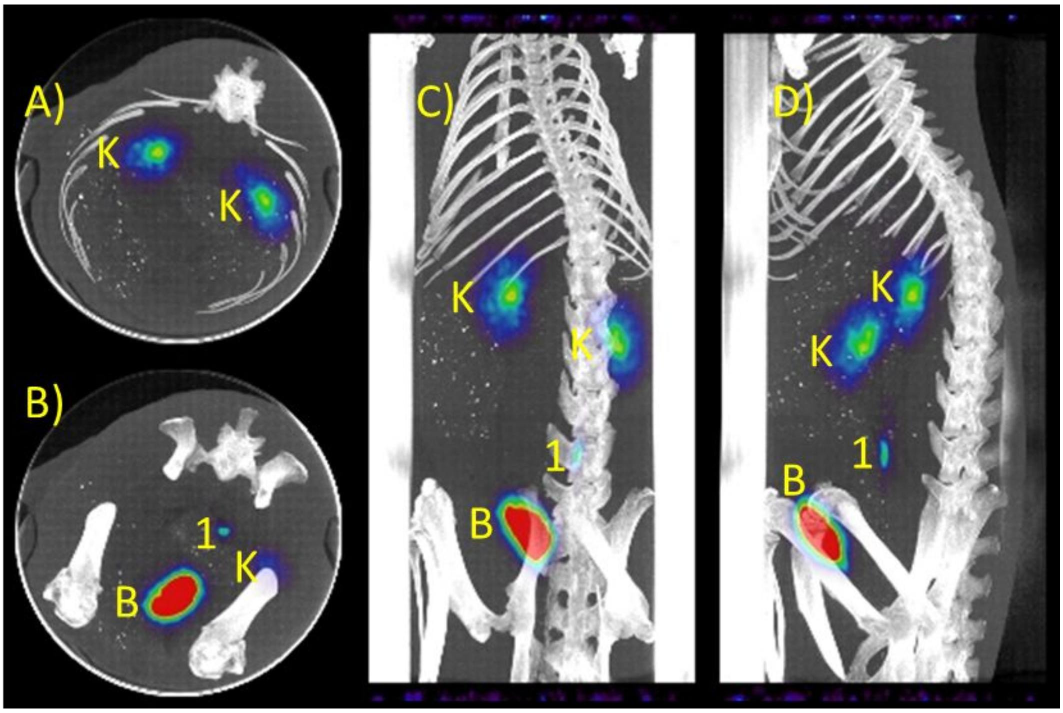

Figure S40: Fused PET-CT scans of a rat 46–66 min post injection with $[^{68}Ga][Ga(\mathbf{Bn_2DT3A})(OH)]^{-}$. A) Transverse projection of upper abdomen. B) Transverse projection of lower abdomen. C) Coronal projection. D) Sagittal projection. Areas of increased uptake are annotated. K = kidneys, B = bladder.

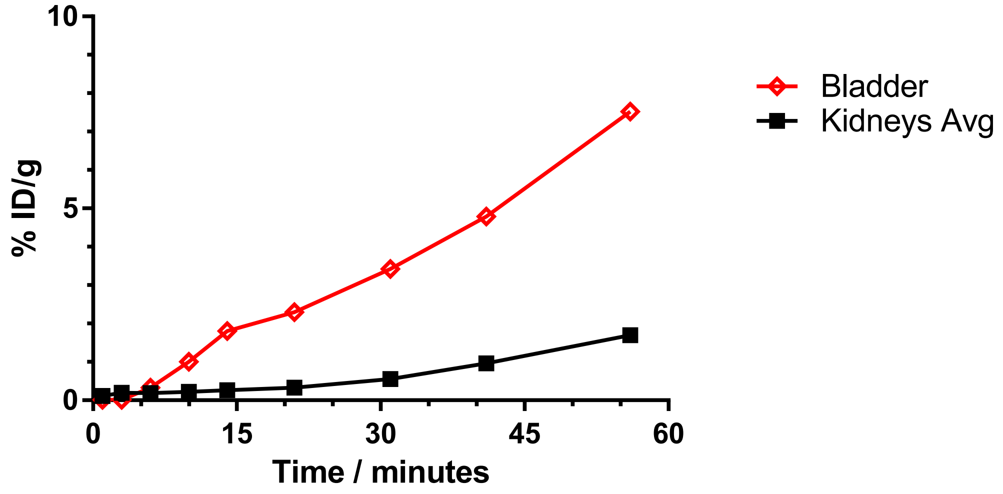

Figure S41: Activity time curves for selected organs following administration of

[68Ga][Ga(**Bn$_2$DT3A**)(OH)]$^-$.

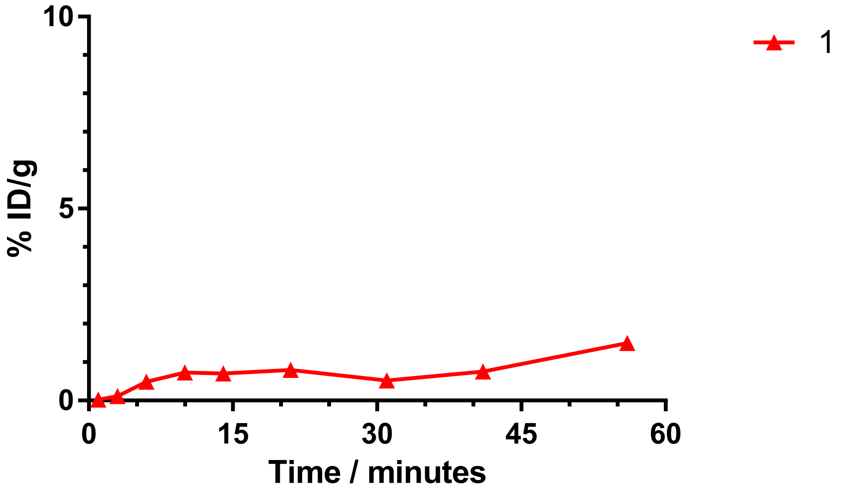

Figure S42: Activity-time curves for additional regions of increased uptake following

administration of [68Ga][Ga(**Bn$_2$DT3A**)(OH)]$^-$.

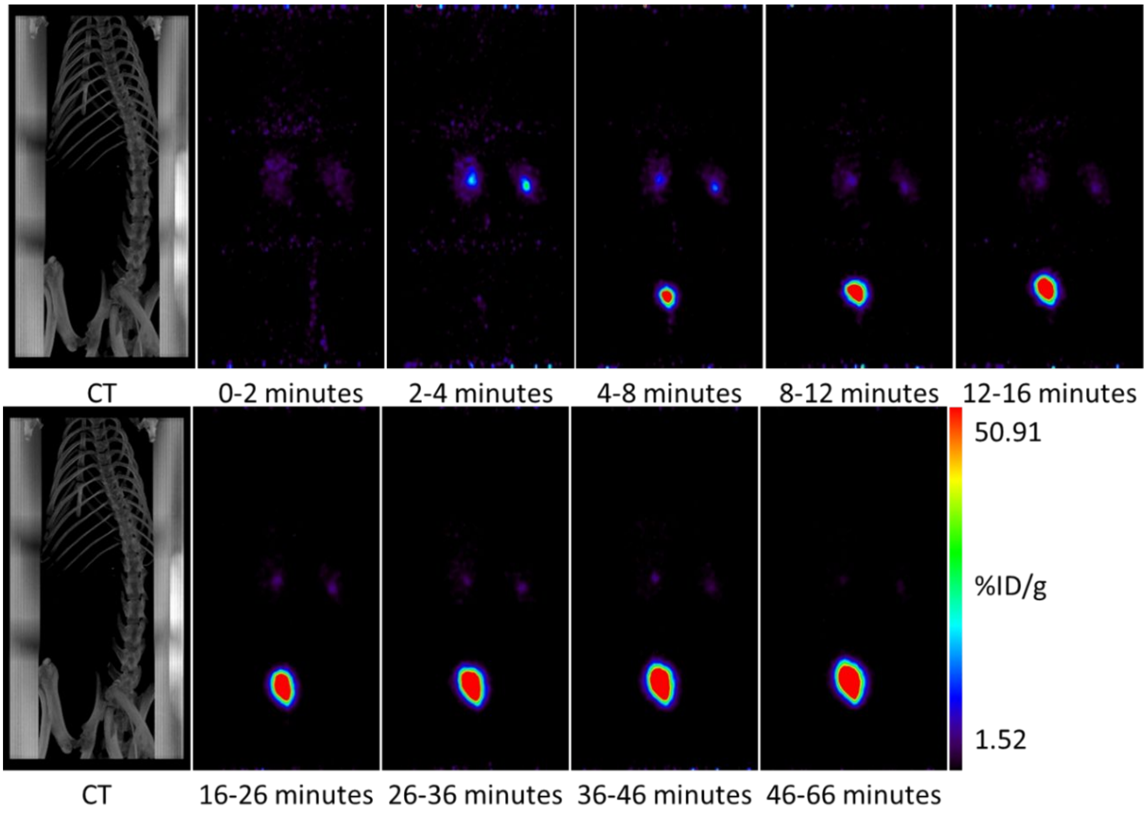

e. [^{68}Ga][Ga(**Bn_2DT3A**)(OH)]$^-$ – 3 bed positions

Figure S43: Coronal PET and CT scans of male rat injected with [^{68}Ga][Ga(**Bn_2DT3A**)(OH)]$^-$

.

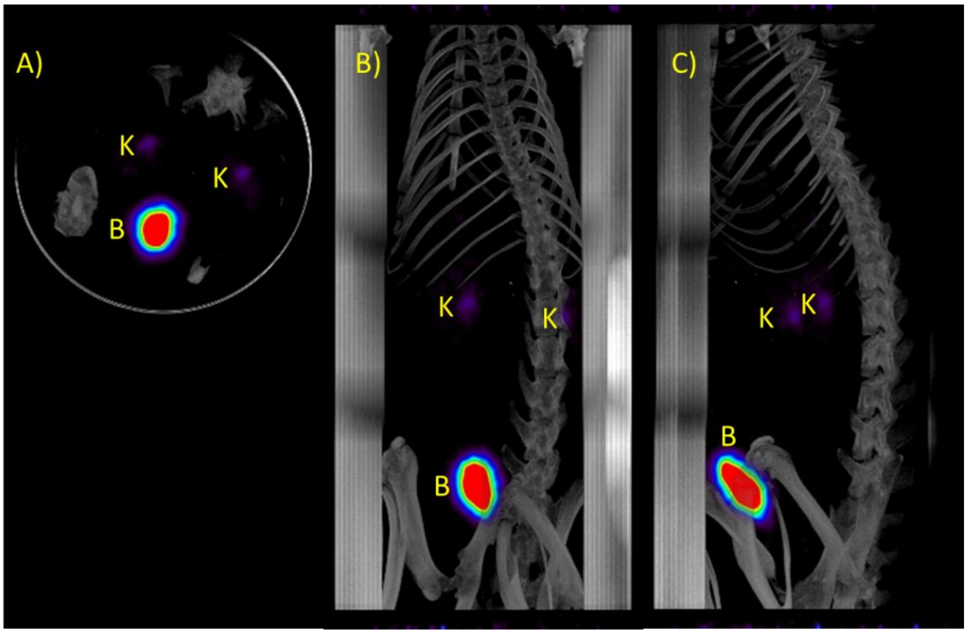

Figure S44: Fused PET-CT scans of a rat 36–46 min post injection with [^{68}Ga][Ga(**Bn$_2$DT3A**)(OH)]$^{-}$. A) Transverse projection of upper abdomen. B) Transverse projection of lower abdomen. C) Coronal projection. D) Sagittal projection. Areas of increased uptake are annotated. K = kidneys, B = bladder.

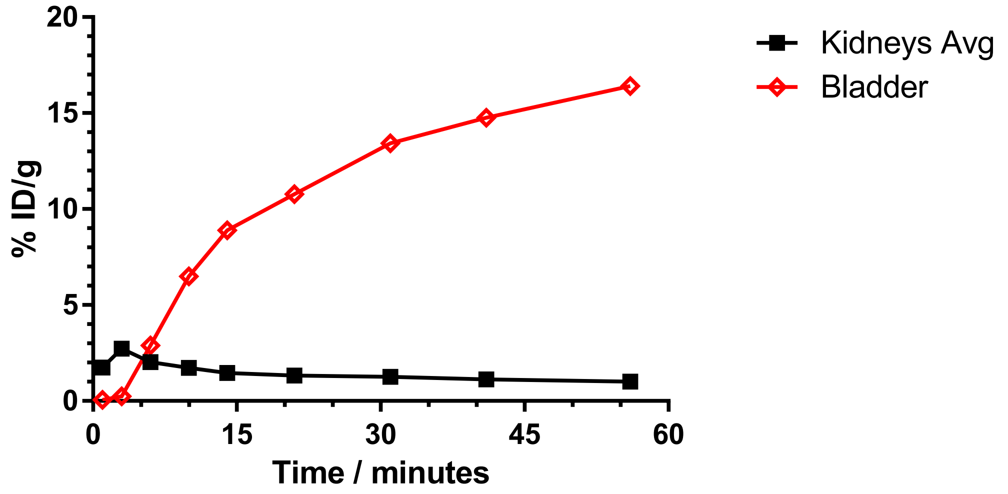

Figure S45: Activity time curves for selected organs following administration of [^{68}Ga][Ga(**Bn$_2$DT3A**)(OH)]$^{-}$.

Table S4: Protonation constants of DTPA and stability constants of its Ga(III) complexes (I = 0.1 M KNO_3, charges are omitted).[1]

Constant	log$K$
HL	10.52
H_2L	8.56
H_3L	4.31
H_4L	2.8
H_5L	2.22
Ga + L = [Ga(L)]	25.11
[Ga(L)] + H = [Ga(HL)]	4.06
[Ga(HL)] + H = [Ga(H_2L)]	1.81
[Ga(L)(OH)] + H = [Ga(L)]	7.1

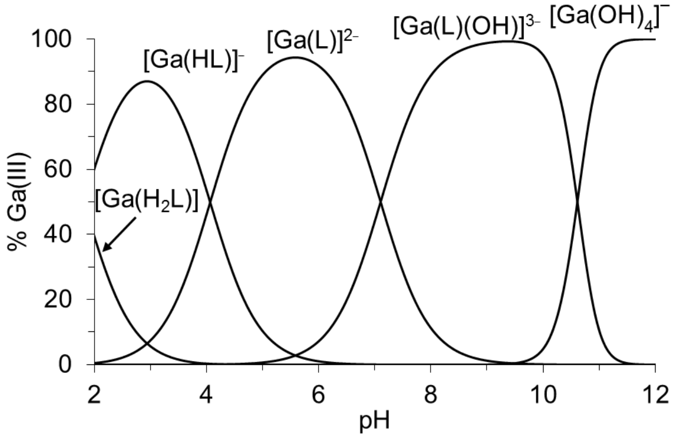

Figure S46: Speciation of Ga^{3+} in solution with DTPA. (T = 25 °C, I = 0.1 M KNO_3, [DTPA] = 4 mM, [Ga^{3+}] = 2 mM).[1]

Table S5: pGa (– logc_{Ga3+}) and p[Ga(OH)$_4$] (– log$c_{[Ga(OH)4]-}$) values for selected chelators. The pGa and p[Ga(OH)$_4$] values were calculated for pH 7.4, c_{Ga} = 1.0 µM, c_L = 10 µM. pGa value is shown for comparison with similar systems published in literature. Formation of tetrahydroxogallate significantly increases the degree of decomplexation, as such p[Ga(OH)$_4$]$^-$ is a more representative measurement for complex conditional stability.

Chelator	pGa	p[Ga(OH)$_4$]
Bn_2DT3A	18.8	5.8
DTPA	22.3	9.3
NOTA	24.8	11.8

7. References

(1) Delgado, R.; do Carmo Figueira, M.; Quintino, S. Redox Method for the Determination of Stability Constants of Some Trivalent Metal Complexes. *Talanta* **1997**, *45* (2), 451–462. https://doi.org/10.1016/S0039-9140(97)00157-4.

(2) Glasoe, P. K.; Long, F. A. Use of Glass Electrodes to Measure Acidities in Deuterium Oxide. *The Journal of Physical Chemistry* **1960**, *64* (1), 188–190. https://doi.org/10.1021/j100830a521.

(3) Sheldrick, G. M. SHELXT - Integrated Space-Group and Crystal-Structure Determination. *Acta Crystallographica Section A: Foundations of Crystallography* **2015**, *71* (1), 3–8. https://doi.org/10.1107/S2053273314026370.

(4) Sheldrick, G. M. Crystal Structure Refinement with SHELXL. *Acta Crystallographica Section C: Structural Chemistry* **2015**, *C71*, 3–8. https://doi.org/10.1107/S2053229614024218.

(5) Táborský, P.; Lubal, P.; Havel, J.; Kotek, J.; Hermann, P.; Lukeš, I. Thermodynamic and Kinetic Studies of Lanthanide(III) Complexes with H_5do3ap (1,4,7,10-Tetraazacyclododecane-1,4,7-Triacetic-10-(Methylphosphonic Acid)), a Monophosphonate Analogue of H_4dota. *Collect. Czech. Chem. Commun.* **2005**, *70*, 1909–1942. https://doi.org/10.1135/cccc20051909.

(6) Försterová, M.; Svobodová, I.; Lubal, P.; Táborský, P.; Kotek, J.; Hermann, P.; Lukeš, I. Thermodynamic Study of Lanthanide(III) Complexes with Bifunctional Monophosphinic Acid Analogues of H_4dota and Comparative Kinetic Study of Yttrium(III) Complexes. *Dalton Transactions* **2007**, *0* (5), 535–549. https://doi.org/10.1039/B613404A.

(7) Baes, C. F.; Mesmer, R. S. The Hydrolysis of Cations. In *Berichte der Bunsengesellschaft & Physikalische Chemie*; Berichte der Bunsengesellschaft & Physikalische Chemie; John Wiley & Sons: New York, London, Syndey, Toronto, 1977; Vol. 81, pp 245–246.

(8) Kývala, M.; Lukeš, I. No Title. *International Conference Chemometrics '95*. Pardubice, Czech Republic 1995, p 63.

(9) Kývala, M.; Lubal, P.; Lukeš, I. No Title. *Spanish-Italian and Mediterranean Congress on Thermodynamics of Metal Complexes (SIMEC 98)*. Girona, Spain 1998.

(10) Gros, G.; Hasserodt, J. Multigram Four-Step Synthesis of 1,4,7-Triazacyclononanes with 2Ra/Rb N-Functionalization Pattern by Starting from Diethylenetriamine.

European Journal of Organic Chemistry **2015**, *2015* (1), 183–187. https://doi.org/10.1002/ejoc.201402821.

(11) Price, E. W.; Cawthray, J. F.; Bailey, G. A.; Ferreira, C. L.; Boros, E.; Adam, M. J.; Orvig, C. H4octapa: An Acyclic Chelator for In-111 Radiopharmaceuticals. *Journal of the American Chemical Society* **2012**, *134* (20), 8670–8683. https://doi.org/10.1021/ja3024725.

(12) Sure, R.; Grimme, S. Corrected Small Basis Set Hartree-Fock Method for Large Systems. *Journal of Computational Chemistry* **2013**, *34* (19), 1672–1685. https://doi.org/10.1002/jcc.23317.

(13) Neese, F. The ORCA Program System. *Wiley Interdisciplinary Reviews: Computational Molecular Science* **2012**, *2* (1), 73–78. https://doi.org/10.1002/wcms.81.
